# *Bacillus subtilis* in defense mode: Switch-like adaptations to protistan predation

**DOI:** 10.1073/pnas.2518989122

**Published:** 2025-09-24

**Authors:** Jordi van Gestel, Byoung-Mo Koo, Vanessa S. Stürmer, Mireia Garriga-Canut, Jonas Wagner, Andrea Zanon, Carol A. Gross

**Affiliations:** ^a^Department of Microbiology and Immunology, University of California, San Francisco, CA 94158; ^b^Developmental Biology Unit, European Molecular Biology Laboratory, Heidelberg 69117, Germany; ^c^Molecular Systems Biology Unit, European Molecular Biology Laboratory, Heidelberg 69117, Germany; ^d^Candidate for joint PhD Degree between European Molecular Biology Laboratory, Heidelberg and Faculty of Biosciences, Heidelberg University, Heidelberg 69117, Germany; ^e^Faculty of Biosciences, Heidelberg University, Heidelberg 69117, Germany

**Keywords:** protistan predation, *Bacillus subtilis*, functional genomics, dictyostelium discoideum, microbial ecology

## Abstract

Single-cell eukaryotic predators in the soil are a primary cause of bacterial cell death. Yet, most functional genomic studies on soil bacteria have been performed without predation, thereby selecting for phenotypes impacting growth rather than survival and biasing our view on the ecological factors driving genomic evolution. Here, we study how predation by the ubiquitous amoebal predator *Dictyostelium discoideum* affects *Bacillus subtilis*’ growth and survival using both a genome-scale mutant screen and de novo evolution of resistance. We show that predation-related genes (many not previously identified) promote survival by enabling filament or aggregate formation, thereby outsizing *D. discoideum* and slowing or preventing ingestion. Importantly, we find that predation resistance is costly, causing a trade-off between growth and survival. *B. subtilis* navigates this trade-off through switch-like adaptations, where cells switch back-and-forth between a slow-growing resistant state and a fast-growing susceptible state. These behaviors are controlled through both genotypic and phenotypic switches, with a central role for the Spo0A phosphorylation cascade, whose ancestral function may have been to evade or slow predation. Taken together, we uncover how the antagonist selection pressure imposed by predation is an important ecological driver of phenotypic heterogeneity in *B. subtilis*.

## Significance

Soil bacteria are part of complex microbial food webs with an abundance of bacterivorous protists, single-cell eukaryotes that live from consuming bacterial prey. Despite the importance of predation, bacteria are often studied in isolation, making their defense strategies elusive. Here, we show that *Bacillus subtilis* evades predation by *Dictyostelium discoideum* through switch-like adaptations that trigger filament or aggregate formation. Defense phenotypes increase survival by avoiding ingestion but come with a growth cost. To navigate this trade-off, both genotypic and phenotypic switching enable *B. subtilis* to switch back-and-forth between a fast-growing susceptible state and slow-growing resistant state. Switch-like adaptations in soil bacteria thus mediate predation evasion much like phase variation promotes immune evasion in pathogens.

Soils are incredibly diverse ecosystems, containing complex microbial food webs with thousands of predator and prey species (1). Bacterivorous protists, single-cell eukaryotes that consume bacterial prey, impose a major predation pressure on bacterial communities (2–5): a single gram of soil easily contains tens of thousands of protistan predators (6–8), each bacterivore can consume hundreds to thousands of bacteria cells per hour (9, 10), and protistan populations can grow rapidly (9, 11). This predation pressure affects the composition of bacterial communities (12–14) and drives rapid evolution of bacterial defenses (15, 16).

Despite its importance, most genome-scale screens on soil bacteria are done in the absence of predators, biasing our view toward growth phenotypes as opposed to survival phenotypes (with exception of pathogens, see refs. 17 and 18). Even *Bacillus subtilis*, one of the best-studied soil bacteria with two genome-wide mutant libraries (19), has never been subjected to unbiased genetic screens under protistan predation. Instead, in both *B. subtilis* (20, 21) and other soil bacteria (22, 23), defense mechanisms are usually studied by examining targeted mutants. These studies revealed a broad range of possible defense mechanisms: Bacteria can, for example, kill predators by secreting biosurfactants (24–26), prevent ingestion by forming biofilms or filaments (27, 28), or resist digestion through sporulation (20, 29).

Genome-scale mutant screens can provide critical insights in genomic adaptation to protistan predation. First, in contrast to case studies that reveal individual mechanisms, genome-scale screens can uncover the full scope of possible defense mechanisms: Which genetic pathways affect predation resistance, which mutations support resistance, and which mutations emerge de novo when bacteria are exposed to predation? Second, genomic-scale screens can reveal systematic trade-offs between growth and survival: Are defense mechanisms costly and, if so, how do bacteria alleviate such costs? Phenotypic trade-offs are at the core of life-history strategies and are critical for understanding how bacteria respond to selection pressures (1, 30–32). Trade-offs also play a central role in regulation (33), allowing bacteria to control costs and benefits by modulating gene expression.

**Fig. 1.**
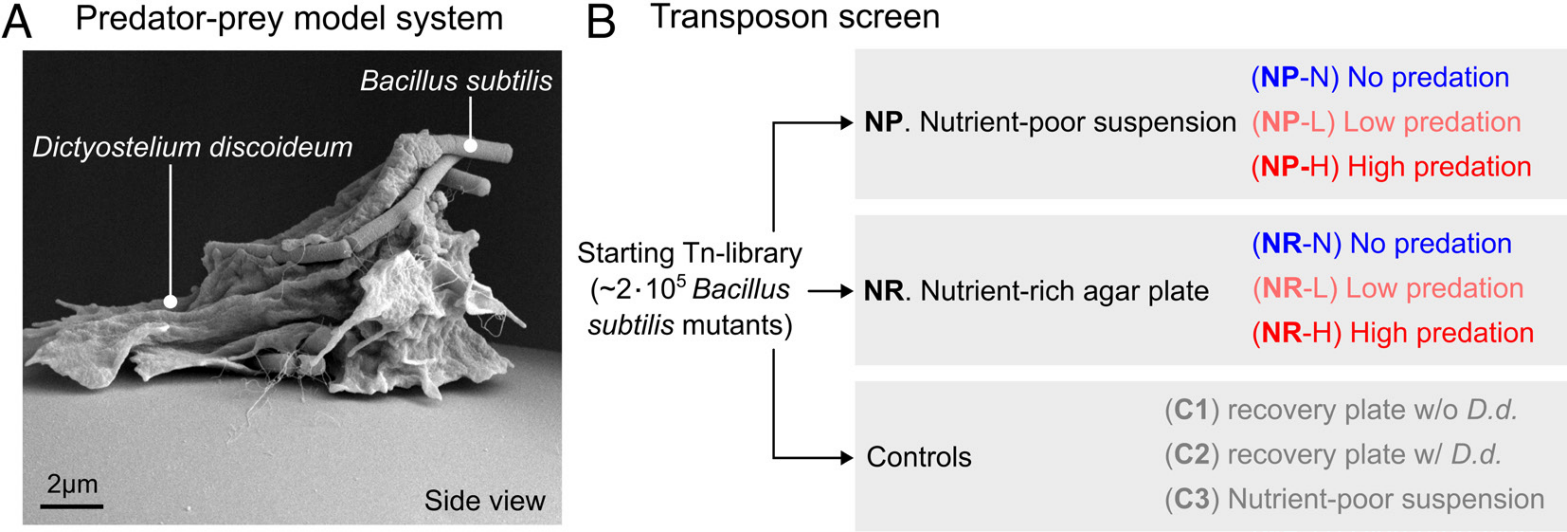
Transposon screen of the predator–prey model system. (*A*) Scanning electron microscopy image of predator–prey model system consisting of *Dictyostelium discoideum* and *B. subtilis*. (*B*) Conditions in transposon screen. Screens starts from a transposon mutant library consisting of approximately 2x10^5^ *B. subtilis* transposon mutants. This library is exposed to no, low, or high predation conditions (*Methods*) in either a (NP) nutrient-poor suspended culturing condition or a (NR) nutrient-rich surface condition. After predation, populations are recovered using recovery plates (*Methods*). As controls, the starting population were also directly plated on recovery plates, either (C1) without or (C2) with *D. discoideum* (*D.d.*). Another control (C3), the starting population was exposed to a starvation condition without any nutrients, as a control for the nutrient-poor suspension.

Here, we perform a genome-wide genetic screen of *B. subtilis* under protistan predation, using the amoebal predator, *Dictyostelium discoideum* (Fig. 1*A*). Amoebal predators are widespread in soils (2) and strongly impact bacterial communities (12)*. D. discoideum* has been used for functional genomic studies before (34, 35) and is a natural predator of *B. subtilis*, which makes it the ideal predator for our study. Using this predator–prey model system, we screen a genome-scale collection of *B. subtilis* mutants for their ability to withstand predation and compare this pool of mutants to those that emerge spontaneously through evolution. Our analyses reveal a large number of mutations affecting predation resistance, three dominant mutational routes leading to resistance, and switch-like adaptations supporting predation defense.

## Results

### Predation Favors Motility, Biofilm, Cell Envelope Biogenesis, and Cell Division Mutants in *B. subtilis*.

To determine which genes support predation resistance, we performed a genome-wide transposon insertion screen with $∼2∙10^{5}$ *B. subtilis* mutants (*SI Appendix*, Figs. S1 and S2). As abiotic conditions can affect predator–prey interactions (23, 36), we exposed our library of transposon mutants to *D. discoideum* in two opposing growth conditions: a nutrient-poor suspension condition (NP) and a nutrient-rich agar plate condition (NR) (Fig. 1*B*). In the NP condition, *B. subtilis* and *D. discoideum* are cultured together in a nutrient-poor growth medium, while shaking, allowing for limited growth of *B. subtilis*. In the NR condition, *B. subtilis* and *D. discoideum* are grown together on nutrient-rich agar plates, allowing for extensive growth of *B. subtilis* and requiring *D. discoideum* to move toward bacteria in space. For each growth condition, we examined three different levels of predation by *D. discoideum* (*Methods*): no predation (N), low predation (L), or high predation (H).

We allowed predation to continue until bacterial populations were visibly cleared at the highest predation level, indicating that few surviving cells remained. In suspension with limited nutrients, bacterial populations were cleared after approximately 6 h (*SI Appendix*, Fig. S3*A*), at which point *D. discoideum* forms aggregates (*SI Appendix*, Fig. S3*B*). On rich media plates, clearance occurred after 3 d, with the onset of fruiting body formation in *D. discoideum* (*SI Appendix*, Fig. S4). To recover bacterial populations, the surviving cells were transferred to a recovery medium and grown at 37 °C, a temperature that kills *D. discoideum*, and then used for transposon sequencing (*SI Appendix*, Fig. S1 and *Methods*). As controls, we included three conditions: two controls where the starting population was immediately transferred to a recovery medium, either in the absence (C1) or presence (C2) of *D. discoideum*, to determine how the recovery stage affects mutant abundances, and a third control where bacterial populations were cultured in a medium depleted of nutrients, to parse out the impact of nutrient starvation in the nutrient-poor condition (C3) (*SI Appendix*, Fig. S5 and *Methods*).

**Fig. 2.**
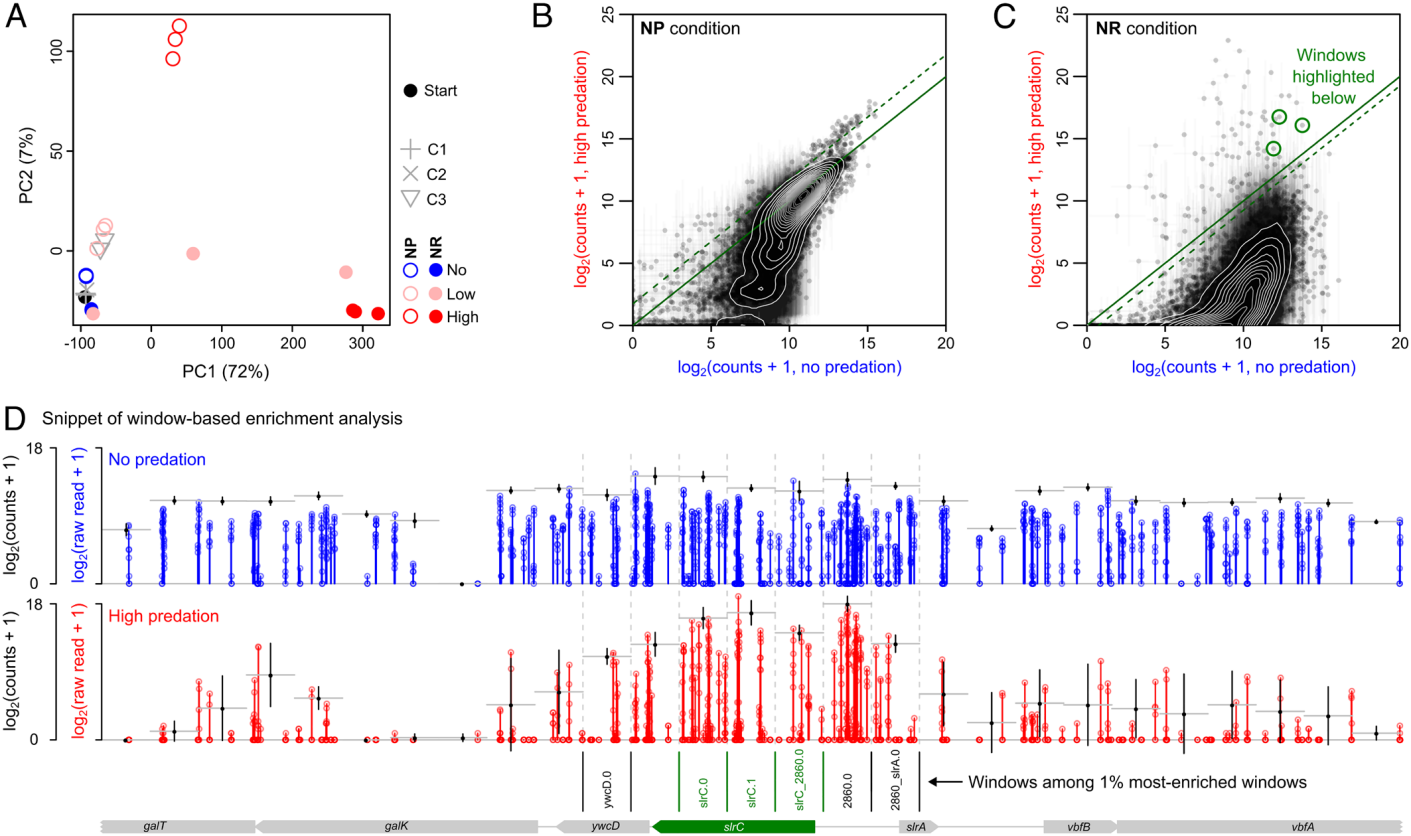
Window-based analysis of Tn-seq screen in the absence and presence of predation. For the window-based analysis, the genome is partitioned in 200 bp windows and mutant abundances are counted on a per-window basis. (*A*) Principal component analysis of mutant abundances across conditions, from starting population (black) to final populations: no predation (blue), low predation (pink), and high predation (red). Open circles show NP growth condition and solid circles show NR growth condition. (*B*) Comparison of mutant abundances in the absence (blue) and presence (red) of predation in nutrient-poor suspension condition (NP). (*C*) Comparison of normalized mutant abundances in the absence (blue) and presence (red) of predation in nutrient-rich surface condition (NR). In (*B*) and (*C*), datapoints show normalized counts (per window); error bars show SD (n = 3); the solid green line shows diagonal, and the dashed green line shows 1% most-enriched windows in high-predation condition (see also *SI Appendix*, Fig. S7). Green circles highlight three of the 1% most-enriched windows that are visualized in (*D*). (*D*) Visualization of window-based analysis for small fraction of the genome. Vertical lines show raw read counts in the absence (blue) and presence (red) of predation. Circles show abundances for individual replicates. Black points and error bars show normalized window-based counts and SD, corresponding to those in (*C*) (horizontal gray lines show 200 bp windows). Windows that are among the 1% most-enriched windows are shown with identifiers (ywdcD.0, slrC.0, slrC.1, slrC_2860.0, 2860.0; 2860_slrA.0). Windows corresponding to *slrC* highlighted in (*C*) are shown in green. See Datasets S2 and S3 for all top-1% enriched windows.

To quantify mutant abundances, we performed both a window-based and a feature-based analyses. For the window-based analysis, the genome of *B. subtilis* was divided in 200 bp windows and mutant abundances were determined for each of them (Fig. 2), whereas in the feature-based analysis, mutants were determined on a gene-by-gene basis (*SI Appendix*, Figs. S5 and S6 and Dataset S1). The 200 bp window is smaller than the length of most protein-coding sequences (median CDS length = 744 bp; 5th to 95th percentile: 174 to 1,886 bp) while spanning a typical intergenic region (median = 115 bp; 5th to 95th percentile: 0 to 342 bp). Quantifying mutant abundances per window therefore provides a more fine-grained view of our data, on which we will focus below, but largely consistent results are obtained with a feature-based analysis (Dataset S1).

We compared overall mutant abundances across samples using a principal component analysis (PCA), where samples with similar mutant counts cluster together (Fig. 2*A*). In the absence of *D. discoideum*, mutant abundances hardly changed relative to the starting population, suggesting that there are no or only minimal growth differences between mutants in nutrient-poor (NP) and nutrient-rich (NR) conditions (see also *SI Appendix*, Fig. S5). In sharp contrast, at high predation levels, we find large changes, orthogonal between both conditions, indicating that different mutants are enriched under predation in suspension (NP) and on agar plates (NR). The largest changes were on agar plates, as shown by the first principal component (i.e., explaining 73% of the variation), which probably results from the longer exposure to predation in this condition (3 d instead of 6 h). At low predation levels, changes in mutant abundances are either small (for the NP condition) or variable across replicates (for NR condition). We will therefore ignore this predation level for the analyses below.

We find tens of mutants enriched under predation in both growth conditions (Fig. 2 *B* and *C* and *SI Appendix*, Fig. S7), although those in suspension (NP) are only weakly enriched, as expected from PCA results (NP, Fig. 2*B*). Mutants on plates show strong enrichment (NR, Fig. 2*C*) going from 0.8 to 90% of the population, underscoring the incredibly strong selection pressure resulting from predation. This selection pressure also results in a strong population bottleneck, where *D. discoideum* consumes most cells in the population, causing substantial variability in mutant abundances between replicates. To estimate the bottleneck sizes, we performed a series of numeric simulations (*SI Appendix*, Text S1). By comparing the depletion of mutants between our simulations and experimental data, we estimate that between 99 and 99.9% of cells are consumed in suspension (*SI Appendix*, Fig. S8) and between 99.9 and 99.99% of cells are consumed on agar plates (*SI Appendix*, Fig. S9). Such strong population bottlenecks increase the risk of jackpot events, where mutants escape predation by chance and become enriched without providing resistance (i.e., false positives) (*SI Appendix*, Text S1). To avoid false positives, we therefore focused our analysis on the 1% most-enriched windows (irrespective of the variability of raw transposon counts inside those windows caused by bottlenecking, Fig. 2*D* and *SI Appendix*, Fig. S7 and Datasets S2 and S3), and validated their selective benefit under predation using targeted follow-up experiments.

As expected from the PCA, different mutations confer predation resistance in each growth condition (*Methods*): Mutants associated with flagella formation and motility (e.g., *sigD, fliK, flgE*) are favored in suspension (NP), while on plates (NR), we find strong enrichment for mutants associated with biofilm formation (e.g., *tapA, sinR, slrC*), cell division (e.g., *divIVA, minJ*), and cell envelope biogenesis (e.g., *pgpB, yodL*). For a complete list of enriched mutants, see Dataset S1. Strikingly, with exception of *sinR*, previously linked to predation defense against ciliates (21), none of the mutants enriched in our transposon screen have been associated with predation defense in *B. subtilis* before, underscoring the importance of performing unbiased genome-scale screens in the context of predation.

**Fig. 3.**
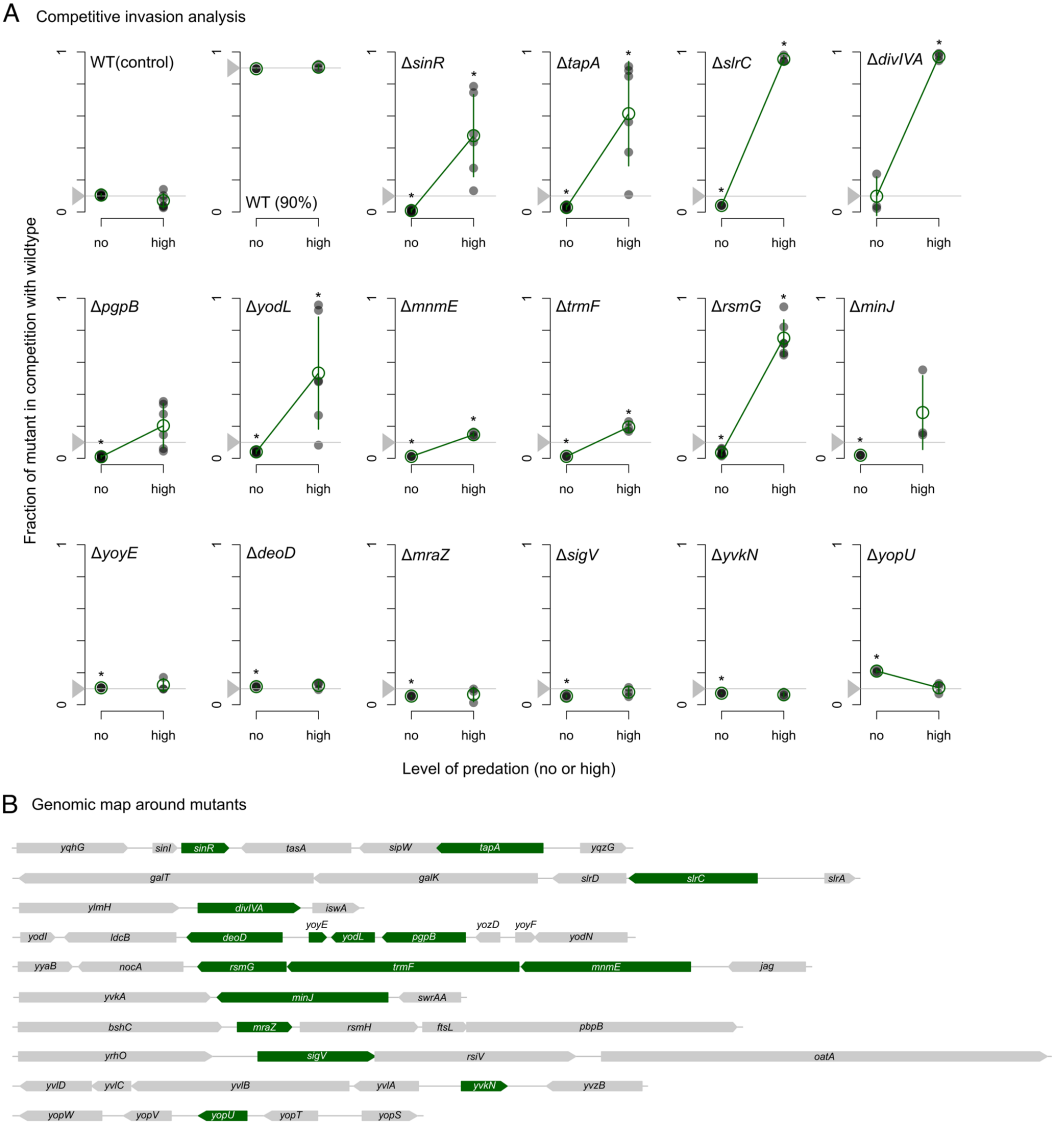
Competition assays between wild-type and mutant strains in the absence and presence of predation. (*A*) Plots show mutant frequencies at the end of competition, as determined by flow cytometry (*SI Appendix*, Fig. S10), in the absence and presence of predation. The horizontal gray line shows initial mutant frequency (10%) before start of competition. Transparent black dots show individual replicates, green circles show mean frequencies, and error bars show SD across replicates (n = 3 to 6). When mutant frequencies significantly differ between no- and high-predation conditions regression lines (green) are shown. The asterisk shows mutant frequencies that significantly deviate from starting frequency (P< <0.05, one-sample *t* test, mu=0.1). (*B*) Genomic maps for each of the mutants in (*A*) to indicate potential polar effects of mutants on the expression of neighboring genes.

To validate our transposon hits, we performed competition assays between mutant and wild-type cells in both the absence and presence of predation. For these assays, we purposely focused on a shortlist of mutants that showed the strongest enrichment in the plate condition ($\log_{2}FC> >3.6$ see *Methods*). After examining the raw reads (Dataset S3), we selected 16 transposon hits, occurring in 10 genomic regions, for our validation experiments (Fig. 3*B*) (these hits showed strong enrichment in both our window-based and feature-based analyses; see Dataset S1). For each hit, we created a full deletion mutant in a *gfp*-expressing background, which we competed against *rfp*-expressing wild-type cells. Starting from a 10% initial mutant frequency, we measured enrichment in the presence and absence of predation (*SI Appendix*, Fig. S10).

Nine of the 16 knockout mutations (Δ*sinR,* Δ*tapA,* Δ*slrC*, Δ*divIVA*, Δ*pgpB,* Δ*yodL,* Δ*mnmE,* Δ*trmF,* and Δ*rsmG*; Fig. 3*A*) showed a significant competitive benefit over the wild-type under predation, and an additional mutant, Δ*minJ*, showed a strong phenotype when tested as a clean deletion with the antibiotic cassette removed (*SI Appendix*, Text S2 and Fig. S11). These 10 adaptive knockout mutations confirm our findings in the transposon screen and underscore the importance of biofilm formation (e.g., Δ*sinR,* Δ*tapA,* Δ*slrC*), cell division (e.g., Δ*divIVA*, Δ*minJ*), and cell envelope biogenesis (e.g., Δ*pgpB,* Δ*yodL*) for predation resistance. Some knockout mutations showed a selective benefit only in the presence of an antibiotic cassette (Δ*mnmE,* Δ*trmF,* Δ*rsmG*, Δ*tapA*), suggesting that their benefits might (in part) rely on the downstream overexpression of genes (*SI Appendix*, Text S2). Similarly, for two of the remaining six genes, Δ*sigV* and Δ*yvkN*, a selective benefit was observed when overexpressing their downstream genes *(oatA* and *yvlA-D*, respectively, *SI Appendix*, Fig. S12). Thus, both knockout effects and downstream gene expression changes were important for predation resistance in our transposon screen.

**Fig. 4.**
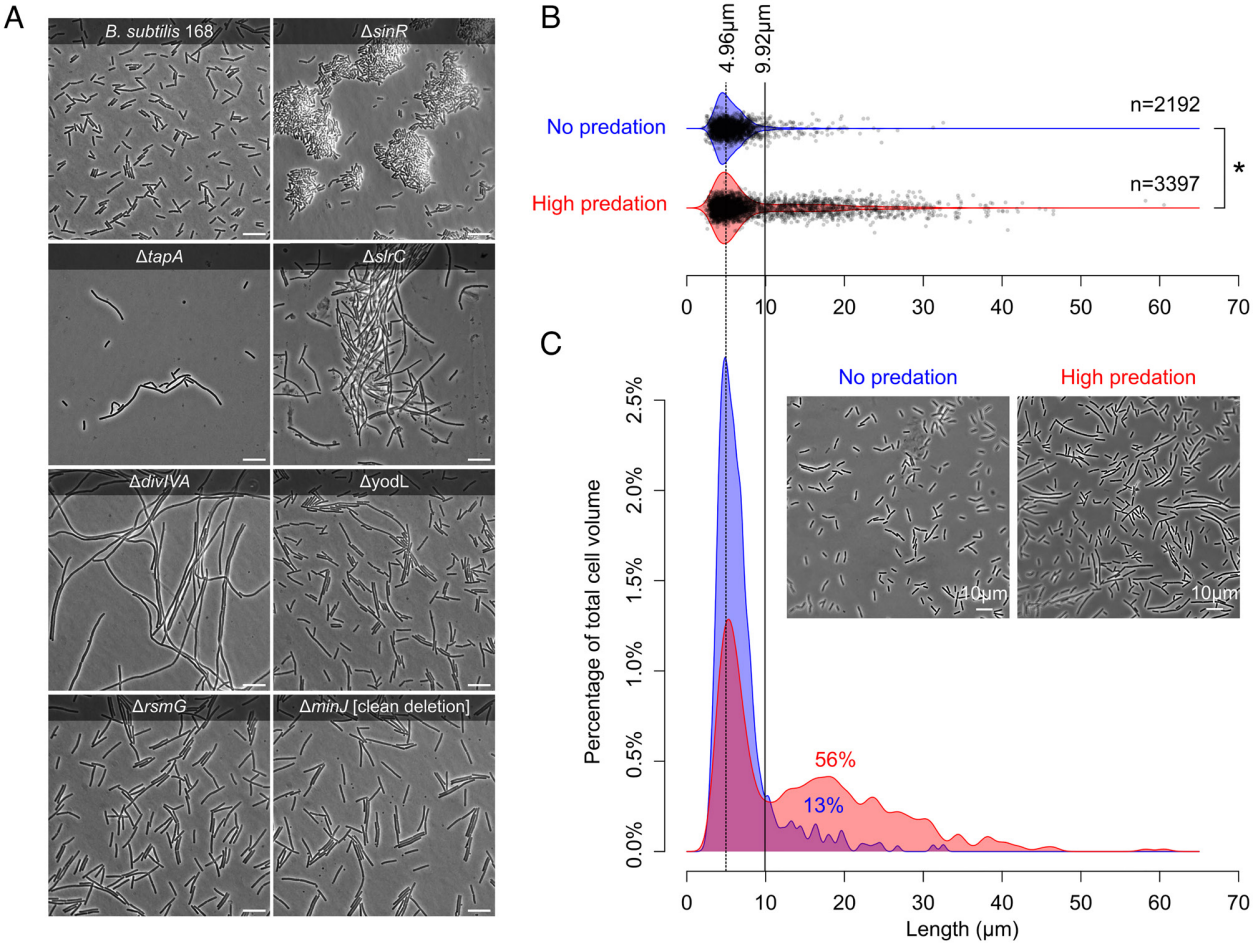
Filamentation and aggregation provide predation resistance. (*A*) Microscopy images of *B. subtilis* wild-type and mutants showing resistant in transposon screen (Fig. 2) and predation assays (Fig. 3*A*). (Scale bar, 10 µm.) (*B*) Quantification of cell/filament lengths, measured from pole-to-pole ends, for the population of transposon mutants from the NP-N and NP-H conditions. With predation, there is a significant increase in cell length (Wilcoxon rank-sum test; $n=5589\mathrm{cells},W=2985117,P< <10^{-15}$). (*C*) Cell volume that is associated with single-cells and filamentous cells. The dotted line shows the average length of single cells (4.96 µm) in both the no and high predation condition. Anything larger than twice the single-cell length is considered filamentous, as shown by the solid line (9.92 µm). Without predation, 13% of the total cell volume is considered filamentous, but with predation, this percentage increases to 56%. Predation pressure thus favors filamentation. See *SI Appendix*, Fig. S13 for all images and Dataset S1 for all measurements.

Microscopic examination revealed a predominance of two phenotypes: Mutants were filamentous (Δ*tapA*, Δ*divIVA*, Δ*yodL,* Δ*rsmG*, and clean deletion of Δ*minJ*) and/or formed aggregates (Δ*sinR* and Δ*slrC*) (Fig. 4*A*). These mutants avoid predation by outsizing *D. discoideum*: They become too big for ingestion and therefore delay or prevent phagocytosis (Movies S1–S4). Given the strong selection pressure that results from predation, we reasoned that the same phenotypic changes should also be apparent on the population level. We therefore directly examined the population of transposon mutants after exposure to predation. Indeed, as expected, we observed a significant increase in filamentation (Fig. 4 *B* and *C* and *SI Appendix*, Fig. S13; Wilcoxon rank-sum test, $n=5589\mathrm{cells},W=2985117,P< <10^{-15}$): 56% of the total cell volume was associated with filamentous growth relative to only 13% in the absence of predation. The presence of filaments in the absence of predation can be attributed to the fact that also wild-type populations heterogeneously induce filamentation, especially during late exponential growth (37–41). We therefore expect that wild-type cells also have a small probability of surviving predation. Together, these results underscore the strong selection pressure that results from predation as well as the broad range of mutants that can mediate resistance.

### Three Mutational Routes Dominate the De Novo Evolution of Predation Resistance.

**Fig. 5.**
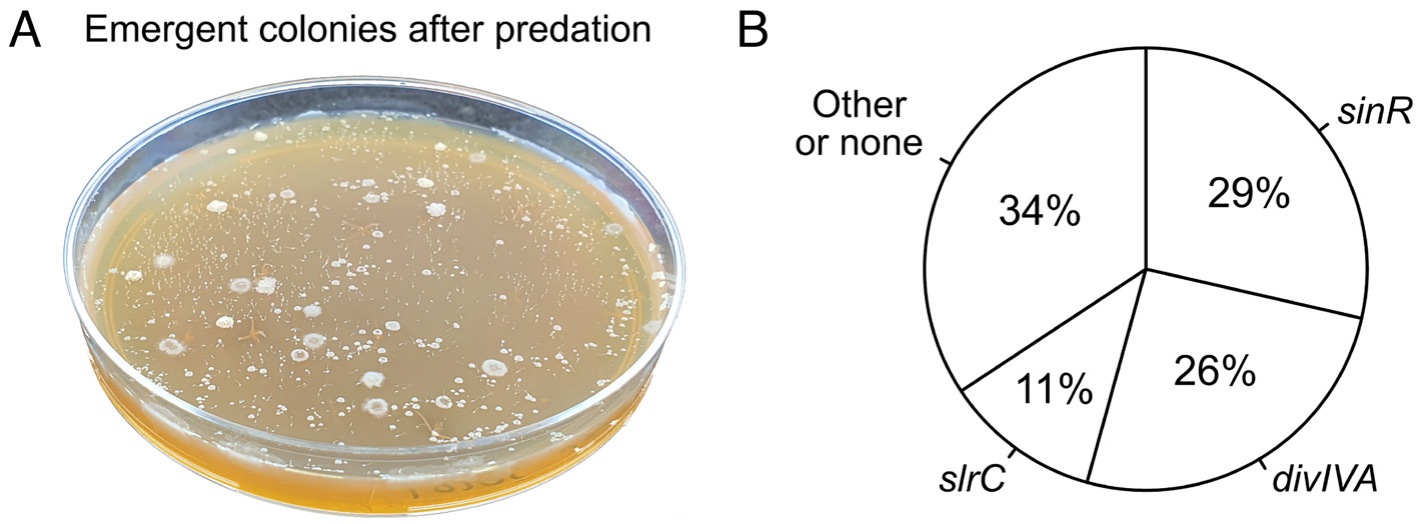
Spontaneous mutants that confer resistance to predation by *D. discoideum*. (*A*) Example plate with emergent colonies—many of which with spontaneous mutants—that are resistant to predation. (*B*) Fraction of colonies that have a mutation in *sinR*, *divIVA,* and *slrC*. Remaining colonies have mutation elsewhere or no mutation at all (e.g., wild-type cells occasionally survive predation as well). See *SI Appendix*, Fig. S15 and Tables S14–S16 for further details.

We next examined which genes are relevant for the spontaneous emergence of resistance. To this end, we exposed *B. subtilis* 168, the partly domesticated lab strain used for our transposon screen, and *B. subtilis* NCIB 3610, a close relative that is undomesticated and produces stronger biofilms (42, 43) to severe predation pressure. We inoculated agar plates with overnight cultures of *B. subtilis* together with *D. discoideum*, and incubated plates at 22 °C until (partly) resistant *B. subtilis* colonies emerged (Fig. 5*A*). The first colonies usually appear within 10 d, showing the rapid speed by which *B. subtilis* populations can adapt to predation pressure. We randomly selected resistant colonies for further inspection. Their phenotypes were strikingly similar to those observed from the transposon screen: Cells were either filamentous or formed aggregates (*SI Appendix*, Fig. S14).

To identify the mutations leading to predation resistance, we sequenced a total of 35 colonies from three independent replicate experiments; one plate with *B. subtilis* 168 and two plates with *B. subtilis* NCIB 3610. We identified a total of 42 mutations (*SI Appendix*, Tables S14 and S15), with most isolates achieving resistance through mutations in one of three loci (Fig. 5*B* and *SI Appendix*, Table S16): *sinR* (29%, n = 10/35 isolates), *divIVA* (26%; n = 9/35 isolates), and *slrC* (11%; n = 4/35 isolates). These mutational hotspots were evident across replicates, irrespective of the *B. subtilis* strain (*SI Appendix*, Fig. S15), and protected against predation by inducing either filamentation and/or aggregation (Movie S5), as seen for the transposon mutants. Besides the three mutational hotspots, we observed several additional mutations (e.g., *minJ*, *pbpB, lexA, ftsZ*; *SI Appendix*, Tables S14 and S15), some of which were not picked up in our transposon screen: *lexA* had insufficient mutational coverage in our screen (i.e., no transposon insertions in *lexA*), while *ftsZ* could not be covered, because it is an essential gene. Both *lexA* and *ftsZ* affect cell division and have never been associated with predation resistance in *B. subtilis* before. LexA represses the SOS response and knockout mutations thereby lead to SOS filamentation (*SI Appendix*, Fig. S16 and Movie S6). FtsZ controls septum formation due to which mutations result in filamentation as well (*SI Appendix*, Fig. S16). In some of the sequenced colonies we did not detect any mutations (*SI Appendix*, Tables S14 and S15), supporting the idea that wild-type filaments can occasionally escape predation as well.

### Antagonistic Selection Predation Defense: Switch-Like Control of Defenses.

**Fig. 6.**
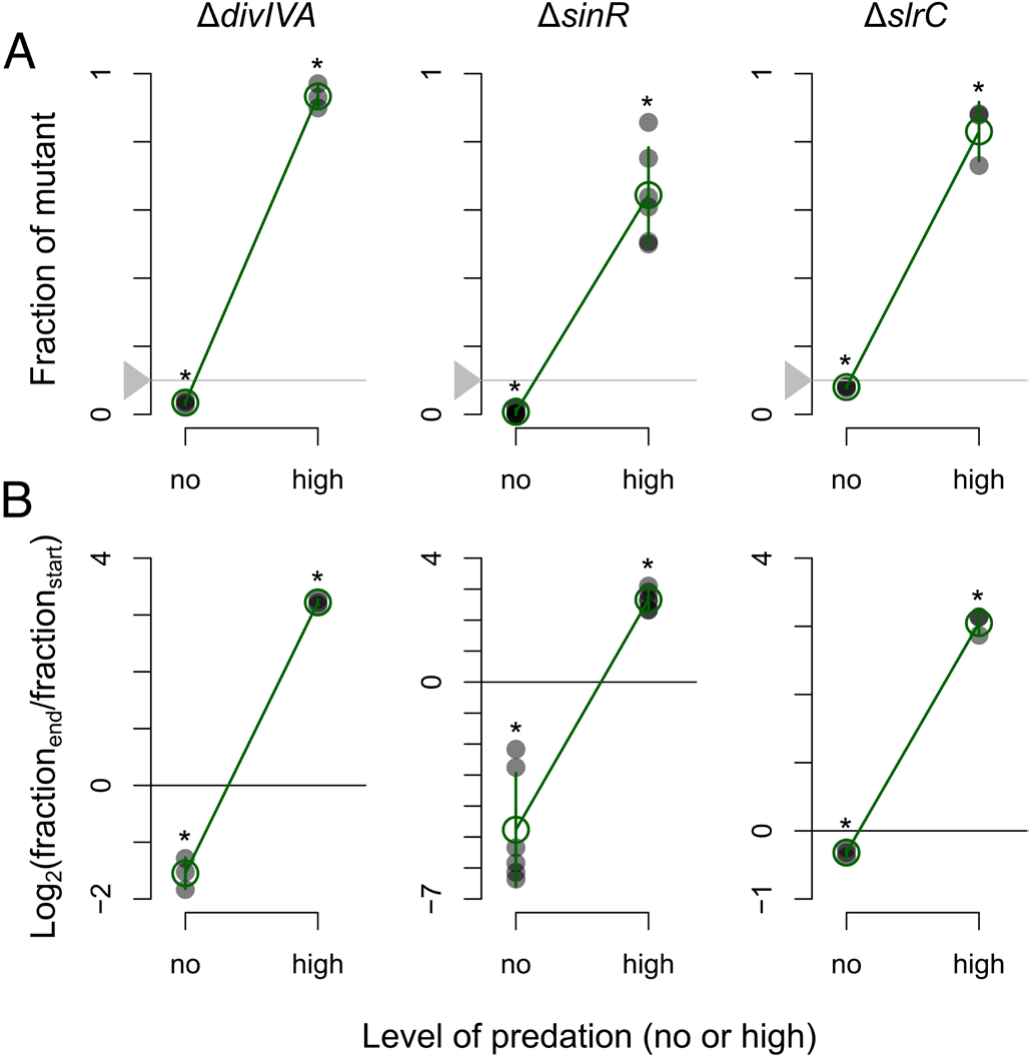
Trade-off between growth and survival: antagonistic selection in the absence and presence of predation. Competition assays between wild-type and ∆*divIVA*, ∆*sinR,* and ∆*slrC* knockout mutants, respectively (clean deletion mutants). (*A*) Fraction of mutant at the end of competition in the absence and presence of predation. The horizontal gray line shows initial mutant frequency at the start of competition (10%). (*B*) Log-transformed frequency of mutant at the end of competition experiment relative to starting frequency. Asterisk show mutant frequencies that significantly deviate from starting frequency [P< <0.05, one-sample *t* test, mu=0.1 in (*A*) and mu=0 in (*B*)]. See Fig. 3 for further details.

Resistance comes with major growth costs. Although mutations in each of the three mutational hotspots (*sinR*, *divIVA,* and *slrC*) have a strong selective advantage under predation, without predation, they are outcompeted by wild-type cells (Fig. 6). The same results were obtained for the other mutants in our transposon screen as well: All resistant mutants have a selective disadvantage in the absence of predation (Fig. 3 and *SI Appendix*, Fig. S11). These results suggest that there is a generic trade-off between growth and survival, leading to strongly antagonistic selection pressures on resistant mutants in the absence versus presence of predation.

**Fig. 7.**
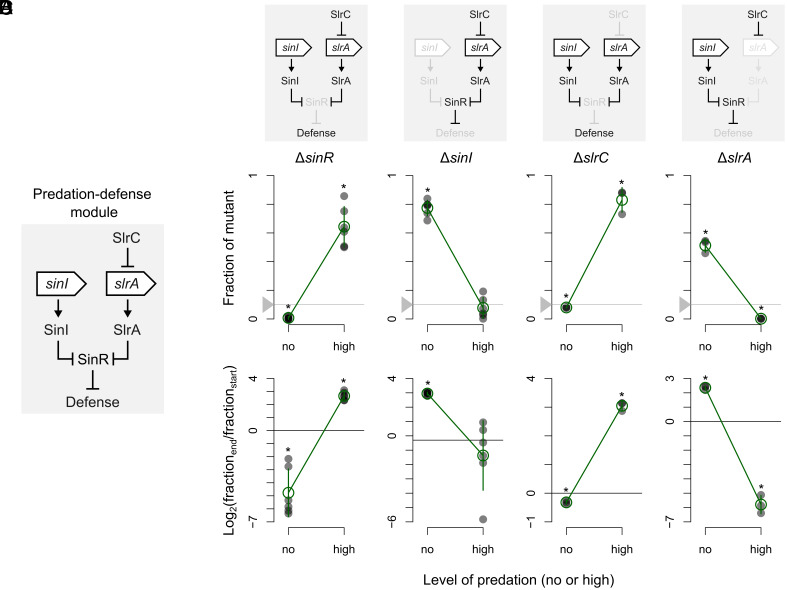
Antagonistic regulation of biofilm-mediated predation defense. (*A*) Simplified overview of predation-defense module underlying biofilm-related defense against *D. discoideum* predation. (*B*) Expected impact of knock-out mutations in *sinR*, *sinI*, *slrC,* and *slrA,* respectively, on expression of predation defense. (*C* and *D*) observed impact of mutation on predation defense. See also caption of Fig. 6. Asterisk show mutant frequencies that significantly deviate from starting frequency [P< <0.05, one-sample *t* test, mu=0.1 in (*C*) and mu=0 in (*D*)]. Mutations that induce biofilm formation are favored under predation, while mutations that lower biofilm formation are selected against.

To study the phenotypic trade-off more closely, we further examined the regulatory pathway underlying biofilm formation (44, 45), as this pathway involves two of the identified mutational hotspots: *sinR* and *slrC*. Inactivating either of them promotes predation resistance by activating biofilm formation, either directly by removing the direct transcriptional repressor of biofilm formation (SinR) or indirectly via a double negative cascade (∆*slrC* prevents repression of SlrA, a direct repressor of SinR) (Fig. 7*A*). In contrast, inactivating either *sinI* or *slrA*, each directly repressing SinR, should increase the amount of SinR and thus prevent costly biofilm formation. We therefore expect the *sinI* or *slrA* knockout mutants to be more susceptible to predation than wild-type cells, but grow faster as a consequence (Fig. 7*B*). Indeed, Fig. 7 *C* and *D* shows that this is the case: In the absence of predation Δ*sinI* and Δ*slrA* outcompete the wild-type, while wild-type cells have a competitive advantage under predation. *B. subtilis* wild-type cells are thus neither fully susceptible nor fully resistant to predation but rather balance growth and resistance; in line with the fact that wild-type cells can filament and have a small probability of surviving predation (Figs. 4 and 5 and *SI Appendix*, Tables S14 and S15).

**Fig. 8.**
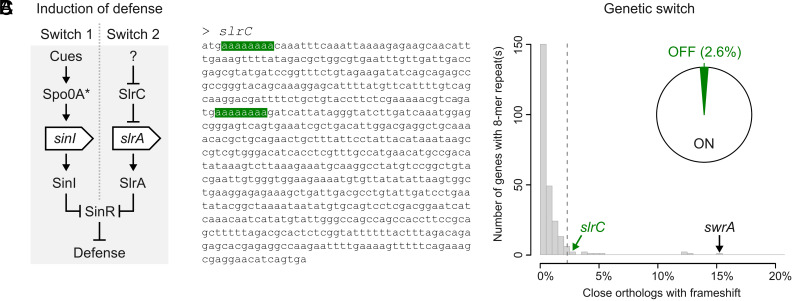
Phenotypic and genotypic switch regulating predation defense in *B. subtilis*. (*A*) Induction of predation defense can take two potential routes: Switch 1, defense is induced by activation of Spo0A through phosphorylation (asterisk), which depends on both environmental cues and quorum-sensing signals. Switch 2, defense is induced by inactivation of *slrC*, as observed among spontaneous mutations in Fig. 5*B* and *SI Appendix*, Table S15. (*B*) Polyadenine 8-mer repeats (green) in CDS of *slrC* that could promote genetic switching through slipped-strand mispairing. (*C*) Number of genes with 8-mer repeats that show frameshift mutations among close orthologs causing premature stop codons. 2.6% of *slrC* orthologs are inactivated due to frameshift mutations in 8-mer repeats (see the pie diagram insert). *swrA* is highlighted as positive control for phase variation.

Given the trade-off between growth and survival, cells should ideally express defenses only in the presence of predation where they are needed, thereby avoiding unnecessary growth costs, leading us to examine how Δ*sinI*/Δ*slrA* and Δ*sinR*/Δ*slrC* are controlled (Fig. 8*A*). *sinI* expression depends on the activation of Spo0A (46), which is controlled by a phosphorylation cascade (43). This well-studied regulatory cascade integrates information from several environmental cues (e.g., starvation cues) as well as quorum-sensing signals (47). Some of these cues may form an indirect proxy for predation risk. For instance, quorum-sensing signals inform cells about the local bacterial density. When this density increases, the predation risk is expected to increase as well, since protists can sense and are attracted by bacteria (48). Quorum-sensing signals might therefore provide indirect information about the predation risk and thereby induce biofilm formation. Interestingly, besides SinR (46), Spo0A also controls DivIVA (49)—linked to predation resistance here as well—and endosporulation. Endospores were previously shown to provide resistance against ciliate predation (20). These results suggest that the core function of the phosphorylation cascade leading to Spo0A activation might be to control predation defenses.

We do not know what controls the SlrC activity, the other route to predation resistance via altered *slrA* expression. However, by examining the *slrC* mutants in our spontaneous mutant screen, we found a putative genetic switch. We observed two independent mutations targeting the same small sequence repeat in *slrC*, both of which led to a premature stop codon (*SI Appendix*, Table S15). *slrC* has two polyadenine 8-mer repeats that could inactivate *slrC* through frameshift mutations (Fig. 8*B*). Such small sequence repeats are often associated with elevated mutation rates due to slipped strand mispairing, leading to phase variation (50–52). In support, frameshift mutations in *slrC* have been reported in *B. subtilis* before (53). The 8-mer repeats in *slrC* could therefore function as a genetic switch, by allowing cells to switch back-and-forth between a fast-growing susceptible state (SlrC active, defenses OFF) and a slow-growing resistant state (SlrC inactive, defenses ON), akin to phase variation expressed by many pathogenic bacteria to evade the immune system during infection (54).

To determine whether *slrC* displays phase variation across genomes, we analyzed all available genomes of *B. subtilis* on NCBI (388 RefSeq genomes). For each gene with an 8-mer repeat (~5% of the genes), we determined the percentage of close orthologs that obtained a frameshift mutation in the nucleotide repeat leading to a premature stop codon (*SI Appendix*, Text S3). For *slrC*, 2.6% of all orthologs had a frameshift mutation, among the top 5% of frameshifts compared to all genes with 8-mer repeats (Fig. 8*C*). For comparison, *swrA*, a gene known to have phase variation (55), 15.5% of all orthologs have a frameshift mutation. *swrA* has a polyadenine 8-mer repeat as well. It was previously estimated that frameshift mutations in *swrA* occur at a frequency of 10^−4^ to 10^−5^ (55), approximately five orders of magnitude higher than *B. subtilis*’ point mutation rate (~10^−10^ mutants per base pair per generation). Interestingly, similar to *slrC*, frameshift mutants of *swrA* cause filamentation as well (56, 57), in this case by lowering *sigD* expression during exponential growth. In support, we observed that *sigD* knockout mutants were strongly enriched in our transposon screen under suspension ($\log_{2}FC=1.98,P< <0.05,$ NP condition; Dataset S1), potentially phenocopying a switch in *swrA* activity (56).

To directly examine whether phase variation in *swrA* is beneficial, we competed wild-type and Δ*swrA B. subtilis* NCIB 3610 cells in the presence and absence of predation (*swrA* is nonfunctional in *B. subtilis* 168). As shown before, Δ*swrA* caused strong filamentation (*SI Appendix*, Fig. S17*C*), which indeed led to a selective benefit under predation (*SI Appendix*, Fig. S17*A*). Most Δ*swrA* filaments were not fully resistant though and could still be consumed by *D. discoideum*, which managed to break filaments at their septa (Movies S7–S9) (58). The selective benefit of Δ*swrA* filaments might therefore primarily be caused by lowering the predation rate rather than by preventing predation. This contrasts with Δ*divIVA* filaments, which have fewer or no septa and can thereby fully block phagocytosis (*SI Appendix*, Fig. S17 and Movies S10–S12).

In summary, phase variation in both *slrC* and *swrA* provides a benefit in the context of predation. Through frameshift mutations, *B. subtilis* cells can switch back-and-forth between a fast-growing susceptible state and a slow-growing resistant state. Since mutations occur stochastically, genetic switches support a bet-hedging strategy, where populations ensure survival when confronted with a sudden exposure to predation, while keeping the costs of resistance in check (i.e., only a small fraction of the population expresses defenses at any moment in time).

## Discussion

Protistan predation imposes a major selection pressure on microbial soil communities, but most functional genomic studies on soil bacteria are done in the absence of natural predators. Here, we performed a genome-scale mutant screen of *B. subtilis* in the context of predation, using the ubiquitous amoebal predator *D. discoideum*. Our screen uncovered tens of genetic mutations that have never been associated with predation defense in *B. subtilis* before. Depending on the growth conditions, these mutations affect cell division, cell envelope biogenesis, or biofilm formation. By filamentation or aggregation, resistant mutants outsize their predator and avoid ingestion. Similar collective defense phenotypes were observed for other bacterial species as well, highlighting their general importance (27, 28, 59–62).

**Fig. 9.**
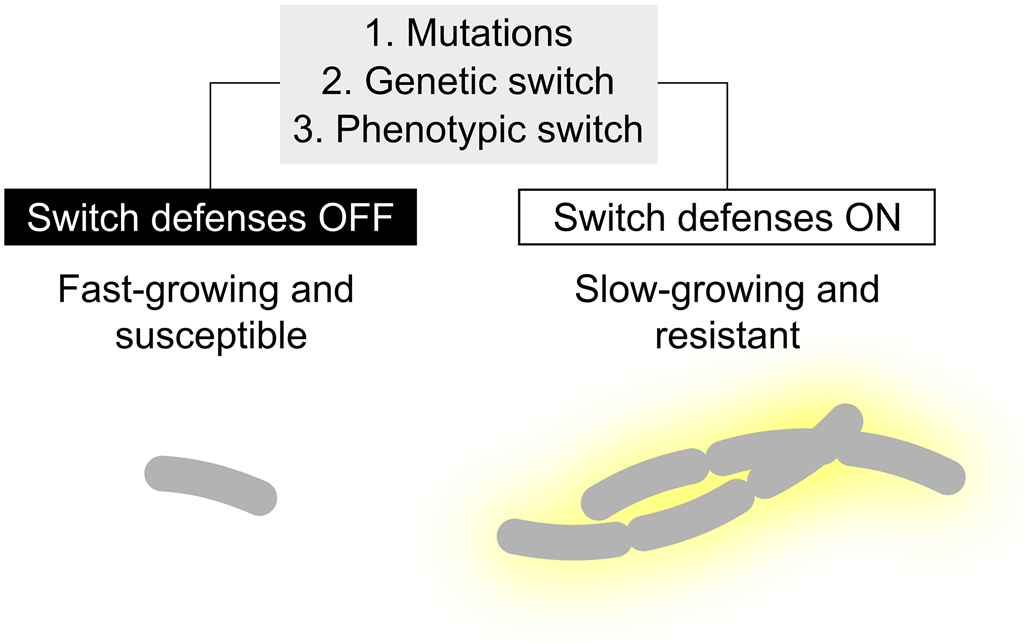
Role of mutations, genetic switches, and phenotypic switches in mediating switch-like adaptation to protistan predation. Cells can evade predation by switching from a fast-growing susceptible to a slow-growing resistant phenotype, where resistant phenotypes either form biofilms and/or show filamentation. Both genetic and phenotypic switches support a reversable bet-hedging strategy, where a fraction of the population can survive in case of an unexpected exposure to predators.

We found that *B. subtilis* defenses can emerge in three possible ways (Fig. 9). First, defenses can evolve de novo. The rapid speed and reproducible manner by which *B. subtilis* evolves resistance, following three dominant mutational routes, suggests that de novo evolution is important under natural conditions as well. Since spontaneous mutations are (mostly) irreversible, de novo evolution locks strains in defense mode. This possibly accounts for the observation that *B. subtilis* populations have a standing genetic variation of genotypes differing in the strength of biofilm formation, whose selective benefits might vary with the local predation pressures (63). Second, defenses can be induced through reversible genetic switches, where cells can switch back and forth between a fast-growing susceptible state and a slow-growing resistant state. We show how small sequence repeats in both *slrC* and *swrA* are sufficient to support switch-like adaptations to predation, much like the role of phase variation in immune evasion by pathogens (50, 54). Finally, defenses can be induced through phenotypic switching. Wild-type *B. subtilis* populations are known to induce both filamentation and biofilm formation heterogeneously (37–40). Similar to genetic switching, this phenotypic heterogeneity could promote survival.

Both genotypic and phenotypic switches could support a bet-hedging strategy (64): By having a fraction of cells expressing defenses, only few cells incur the costs of resistance, while the population can survive an unexpected exposure to predation. Bet-hedging strategies generally evolve under strong antagonistic selection pressures that occur either infrequently or unexpectedly, making it difficult to (solely) rely on phenotypic response strategies (65). We show that predation imposes such strong antagonistic selection pressures: >99% of *B. subtilis* cells are eradicated under predation, while predation defenses come with major growth costs. Other soil bacteria are likely exposed to similar antagonistic selection pressures as well (66), suggesting that bet-hedging strategies might be widespread. When populations are exposed to fluctuating predation pressure, bet-hedging improves the reproductive output of a lineage as cells propagate through various episodes of predation (67, 68). This reproductive output is usually quantified through the geometric mean fitness and is determined by the multiplicative process of division over time (64, 69). Thus, an important future challenge lies in determining whether and how genotypic and phenotypic switching affects the geometric mean fitness of *B. subtilis* in natural soil environments, in exposure to natural fluctuations in predation pressure (70).

Our study centralizes the role of Spo0A in regulating predation defenses. Spo0A controls three types of predation defenses: aggregation (i.e., biofilm formation), filamentation, and endosporulation. The importance of the first two defenses were demonstrated in this study, while Klobutcher and colleagues (20) previously highlighted the relevance of endosporulation. Although the regulatory link between biofilm formation and sporulation has been known for years, why *B. subtilis* uses the same regulatory cascade to control both phenotypes remained puzzling. Our work suggests that Spo0A enables *B. subtilis* to express defenses in an escalating manner: Cells induce biofilm formation and filamentation under growth permissive conditions and switch to endosporulation when conditions deteriorate (71, 72). We expect that by expressing defenses when predation risk is high, the regulatory cascade controlling Spo0A activity navigates the trade-off between growth and survival.

The importance of collective defenses in *B. subtilis*, like aggregation and filamentation, further supports the long-standing hypothesis that predation is a major ecological driver for the emergence of nascent multicellularity (73, 74). Previous evolution experiments on both bacteria (32, 75, 76) and eukaryotic microbes (77–79) showed that predation leads to the rapid emergence of multicellular phenotypes. Our study corroborates the strong selective advantage of multicellularity under predation and also suggests a reason for its rapid emergence. The genomic substrate for evolving multicellular predation defenses is particularly large: Tens of mutations in both essential and nonessential genes give rise to multicellularity either by mediating incomplete cell division leading to filamentation or by allowing for aggregation through matrix production. This could explain why multicellular defenses evolve more readily than other defenses (22) and why widely different model systems evolve strongly convergent multicellular phenotypes in exposure to predation.

The benefits of multicellularity are apparent within the smallest collectives. Although biofilm mutants like ∆*sinR* and ∆*slrC* are usually studied at the macroscopic scale (42, 43), in the context of predation, benefits emerge when aggregates and filaments are only a few tens of microns in size. Micron-scale properties can in fact strongly influence predation kinetics: We show that filaments with septa (e.g., Δ*swrA*) can, for example, be broken by *D. discoideum*, while those without cannot (e.g., Δ*divIVA*). Filaments might thus either delay or entirely block ingestion. Although small morphological features, like the presence of septa, might seem irrelevant when studying *B. subtilis* in isolation, they can decide between life and death under predation. This highlights the importance of studying bacteria in context of their natural interaction partners and at spatial scales under which interactions unfold.

By examining predation resistance of *B. subtilis* against amoebal predation, we took a first step in embracing the complexity of natural food webs. Bacteria are however exposed to a much broader range of predators, including ciliates, flagellates, and many nematodes (8). We therefore advocate for increased efforts in bridging the fields of systems biology and microbial ecology, with the ultimate goal of understanding how ecological interactions shape genomic evolution (1).

## Methods

See *SI Appendix*, Text S3 for a detailed description of the methods and Zenodo (80) (https://doi.org/10.5281/zenodo.14871691) for all supplementary data files (Datasets S1–S4 and Movies S1–S12).

### Strains and Culturing.

For cloning and standard culturing, *B. subtilis* (strains 168 and NCIB 3610) was grown on LB at 37 °C. For selective plating, antibiotics were used at standard concentrations (*SI Appendix*, Text S3). For predation experiments, *Dictyostelium discoideum* NC4 (DBS0304666; dictyBase) was typically precultured on LPB plates with *Escherichia coli* B/r (DBS0305924; dictyBase). See *SI Appendix*, Tables S1–S10 for strain lists, consumables, and media compositions. The mariner Transposon library in *B. subtilis* 168 ($∼2∙10^{5}$ transposon insertion mutants) was kindly provided by Alan Grossman and was generated as described in ref. 81. The transposon library was grown to a density of ~$5∙10^{8}$ cells/mL (OD_600_=1) and mixed 50:50 (v/v) with $0,{6.25∙10}^{5}$ or $10^{7}$ *D. discoideum* cells/mL, creating the no (N), low (L), and high (H) predation levels, respectively. These mixed populations were cultured in either a nutrient poor suspension (NP) condition consisting of SorMC buffer with a 2.5% (v/v) LB spike-in or a nutrient-rich agar plate (NR) condition consisting of SM/5 agar plates. Predation was allowed to happen until *B. subtilis* populations were visibly cleared at the high predation level, which took about 6 h for the NP condition and 3 d for the NR condition. After predation, all *B. subtilis* populations were recovered by transferring them to recovery plates (LB plates), which were incubated for 5 to 6 h at 37 °C (a temperature where *D. discoideum* dies). Through this recovery step, we could generate sufficient biomass for transposon library preparation.

### Transposon Library Preparation and Analysis.

Genomic DNA (gDNA) from recovery plates was extracted, digested with MmeI, and ligated to barcoded adapters (*SI Appendix*, Fig. S1). Libraries were PCR-amplified, size-selected via gel excision, and sequenced on an Illumina HiSeq 4000 platform (single-end reads, dual-barcoding). A total of 24 libraries were sequenced across five lanes (*SI Appendix*, Table S11). The complete dataset is publicly available via the European Nucleotide Archive (ENA) under accession number PRJEB85855. Reads were subsequently mapped to the *B. subtilis* 168 genome (RefSeq, GCF_000009045.1, NC_000964_3, Assembly ASM904v1) using Bowtie 1.3.1, followed by a conversion to wig files and normalization. Mutant abundances were quantified either through a window-based analysis (using 200 bp windows) or a feature-based analysis, where windows and features with low coverage were excluded (< <5% of the median count values). The count matrices are provided in Dataset S1. To determine the impact of population bottlenecks caused by predation, we also simulated the expected read counts based on the empirical input distribution (i.e., starting population, *SI Appendix*, Texts S1 and S3). These simulations were used to estimate the fraction of cells that were consumed under high predation levels and guide the interpretation of the observed mutant enrichments (*SI Appendix*, Figs. S8 and S9).

### Spontaneous Mutant Isolation.

To screen for spontaneous mutants leading to predation resistance, we incubated both *B. subtilis* 168 and *B. subtilis* NCIB 3610 with *D. discoideum* NC4 on nutrient-rich SM plates, until resistant colonies emerged (~10 to 15 d). gDNA of resistant isolates was extracted using Promega’s Wizard Genomic DNA Purification Kit (Ref A1120, Promega) according to the manufacturer’s instructions (*SI Appendix*, Text S3). Library construction and whole-genome sequencing were performed by Microbial Genome Sequencing Center (MiGS, Pittsburg, USA; current renamed as SeqCenter). Genomes were sequenced using Illumina NextSeq 2000 platform with 151 bp paired-end reads. All sequencing data are publicly available on the European Nucleotide Archive (ENA) database, accession number: PRJEB85855. Reads were mapped against associated reference genomes, *B. subtilis* 168 (RefSeq, GCF_000009045.1) and *B. subtilis* NCIB 3610 (RefSeq, GCF_002055965.1), using Breseq 0.35.4 (82, 83), and all identified mutants were evaluated manually using the Integrated Genome Browser, IGVTools 2.4.19 (84). Mutants are listed in *SI Appendix*, Tables S13–S15.

### Strain Construction and Competition Assays.

To validate Tn-seq hits, we performed competition experiments between mutant and wild-type cells. Clean knockout mutants were generated using the whole-genome knockout library of *B. subtilis* 168 (19) in fluorescent background strains (see *SI Appendix*, Table S9 for details). Further cloning details are provided in *SI Appendix*, Text S3, including information about oligos and integration plasmids (*SI Appendix*, Tables S8 and S10). For *B. subtilis* NCIB 3610, mutants were generated by transforming a ∆*comI* background strain [*SI Appendix*, Table S9, (85)] with the gDNA from a knockout strain of interest, obtained from the whole-genome knockout library (19). All mutants were confirmed through PCR and Sanger sequencing.

Competition experiments were performed with mixed populations consisting of 90% wild-type and 10% mutant cells with complementary fluorescent reporters, allowing us to quantify the competitive fitness of mutants using flow cytometry (BD LSR II Flow Cytometer; see *SI Appendix*, Fig. S10). For competition experiments in the NP condition, cells were competed on SM/5 agar plates for 3 d in either the presence or absence of predation, subsequently allowed to recover on LB suspension at 37 °C and analyzed using flow cytometry to determine mutant frequencies. All flow cytometry data are publicly available through Zenodo (10.5281/zenodo.14871691). To visualize single-cell phenotypes of *B. subtilis* wild-type and mutants, we also performed widefield microscopy using agarose pads. *B. subtilis* was grown overnight in LB, back diluted and grown until an OD_600_ between 1 and 2. Cells were spotted on agarose pads either with or without *D. discoideum* and imaged using a Nikon Ti2-E inverse microscope. For further details on imaging settings and analysis, see *SI Appendix*, Text S3.

## Supplementary Material

Appendix 01 (PDF)

## Data Availability

Sequencing data and Flow cytometry data have been deposited in ENA and Zenodo (PRJEB85855 and 10.5281/zenodo.14871691) (80, 86).
