# Supplementary material for: Bacillus subtilis in defense mode: Switch-like adaptations to protistan predation
Source: Proc Natl Acad Sci U S A. 2025 Sep 24;122(39):e2518989122. doi: 10.1073/pnas.2518989122 (PMC12501158; doi:10.1073/pnas.2518989122)
Supplement: Supplementary file 1 — Appendix 01 (PDF) [file pnas.2518989122.sapp.pdf]

Supplementary information

Bacillus subtilis in defense mode: switch-like adaptations to protistan predation

Jordi van Gestel, Byoung-Mo Koo, Vanessa Stürmer, Mireia Garriga-Canut, Jonas Wagner, Andrea Zanon & Carol Gross

Table of content Page

**Figures S1-S17**	2
**Movies S1-S12**	19
**Tables S1-S16**	20
**Text S1.** Exploring the impact of population bottlenecks	26
**Text S2.** Competition assays with clean deletion mutants	27
**Text S3.** Detailed methods	27

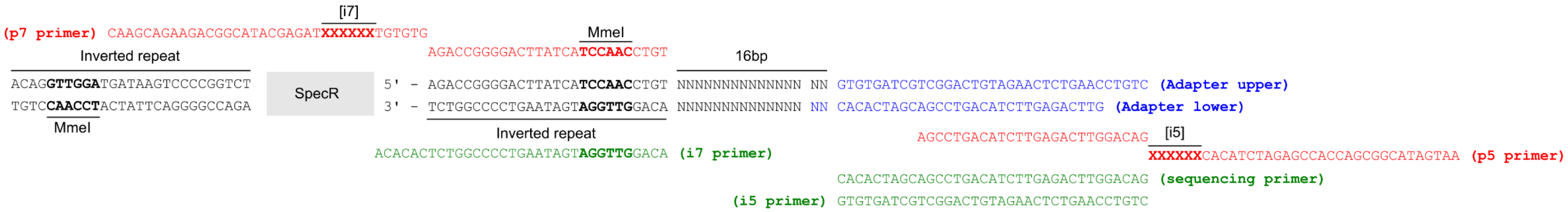

Figure S1. Primer design for transposon sequencing. After digestion with MmeI, adapters (blue) are ligated to DNA and the 16bp genome region is PCR-amplified using the p5 and p7 primers (red). These primers immediately include the sequencing barcodes [i5] and [i7]. Illumina sequencing is done using i5 primer, i7 primer and sequencing primer (green). See Table S8 for overview of oligos and Methods for further details.

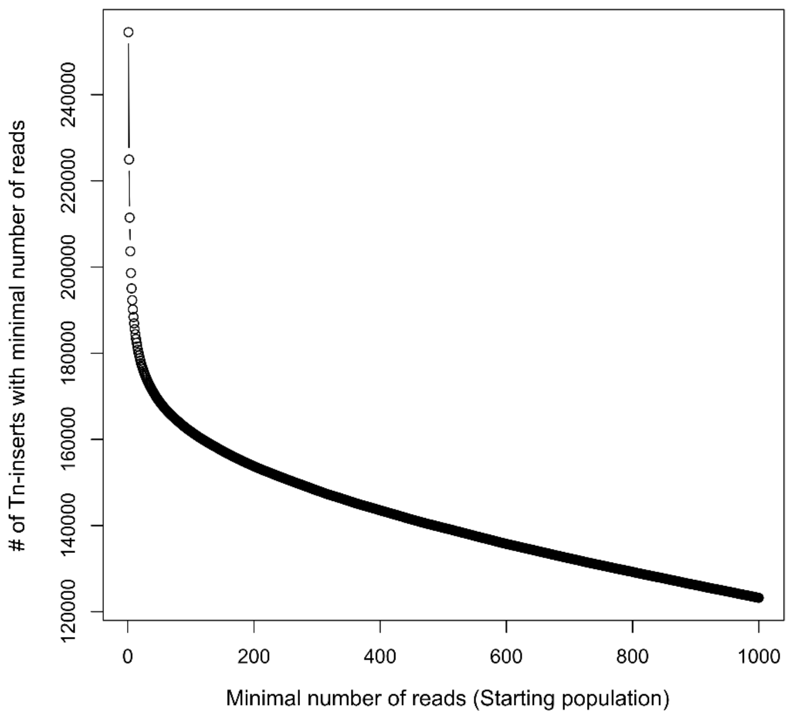

Figure S2. Read distribution across Tn-insert mutations. Number of inserts with minimal number of read in starting population. Approximately 250.000 Tn-inserts were observed with at least 1 read, with an elbow in the distribution around 170.000 Tn-inserts. Tn-inserts that were associated with low read abundances in the starting populations were ignored in our analyses (see Methods).

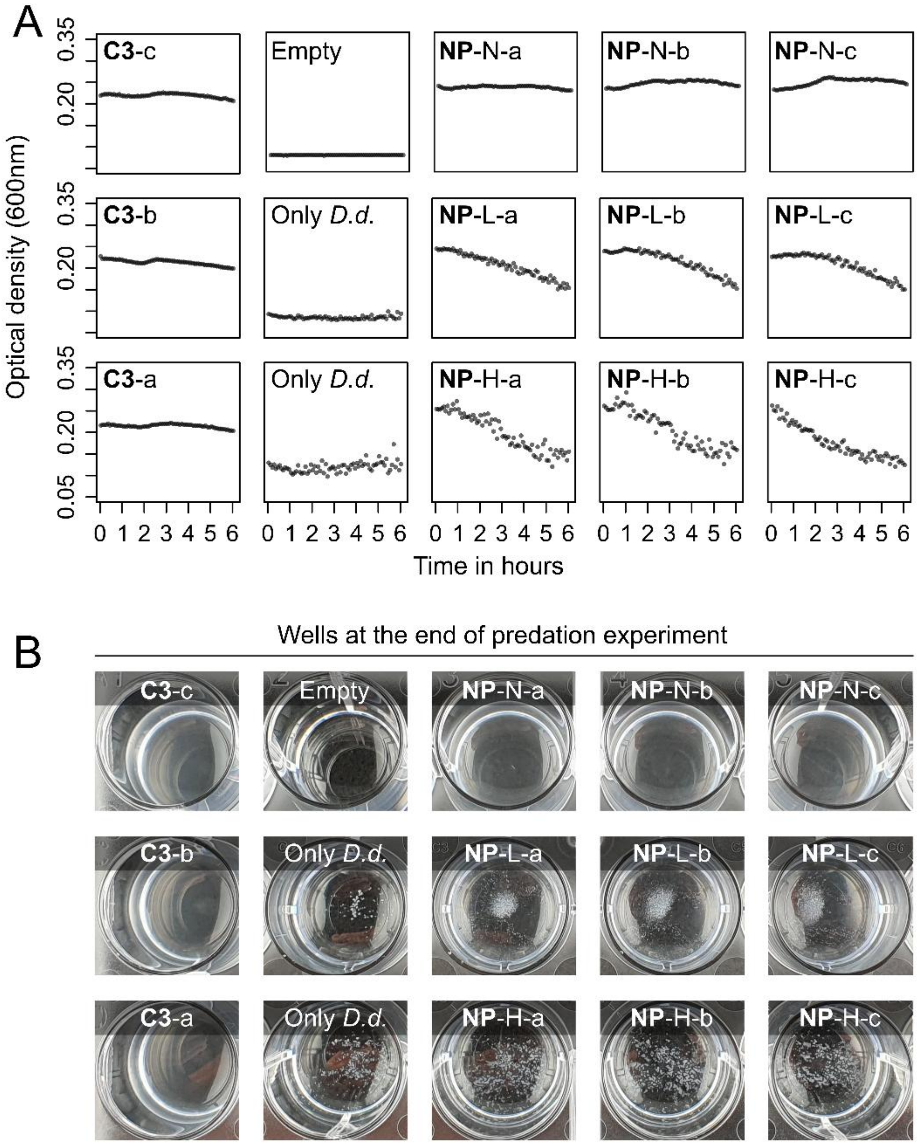

Figure S3. Predation dynamics in nutrient-poor suspended (NP) condition. (A) Optical density (OD_{600}) of population with transposon mutants in suspended condition over time course of ~6 hours: no predation (NP-N), low predation (NP-L), high predation (NP-H) and starvation controls (C3). As reference we also show empty well and well with low or high density of *D. discoideum* (*D.d.*) only. (B) Wells at the end of predation experiment. Clearance of bacterial cell suspension under high predation condition is apparent, and *D. discoideum* cells aggregate on bottom of the wells.

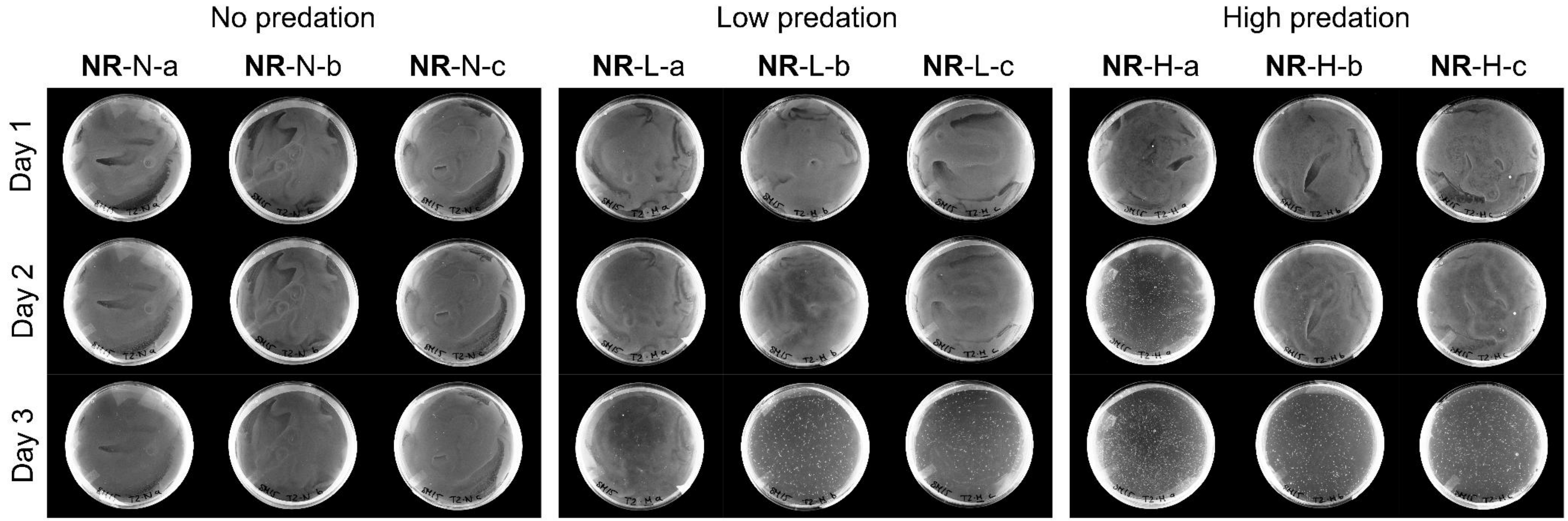

Figure S4. Predation dynamics in nutrient-rich surface (NR) condition. SM/5 agar plates over the time course of three days: no predation (NR-N), low predation (NR-L), high predation (NR-H). After three days in the high predation condition, *B. subtilis* populations are cleared from the surface, triggering fruiting body formation in *D. discoideum*.

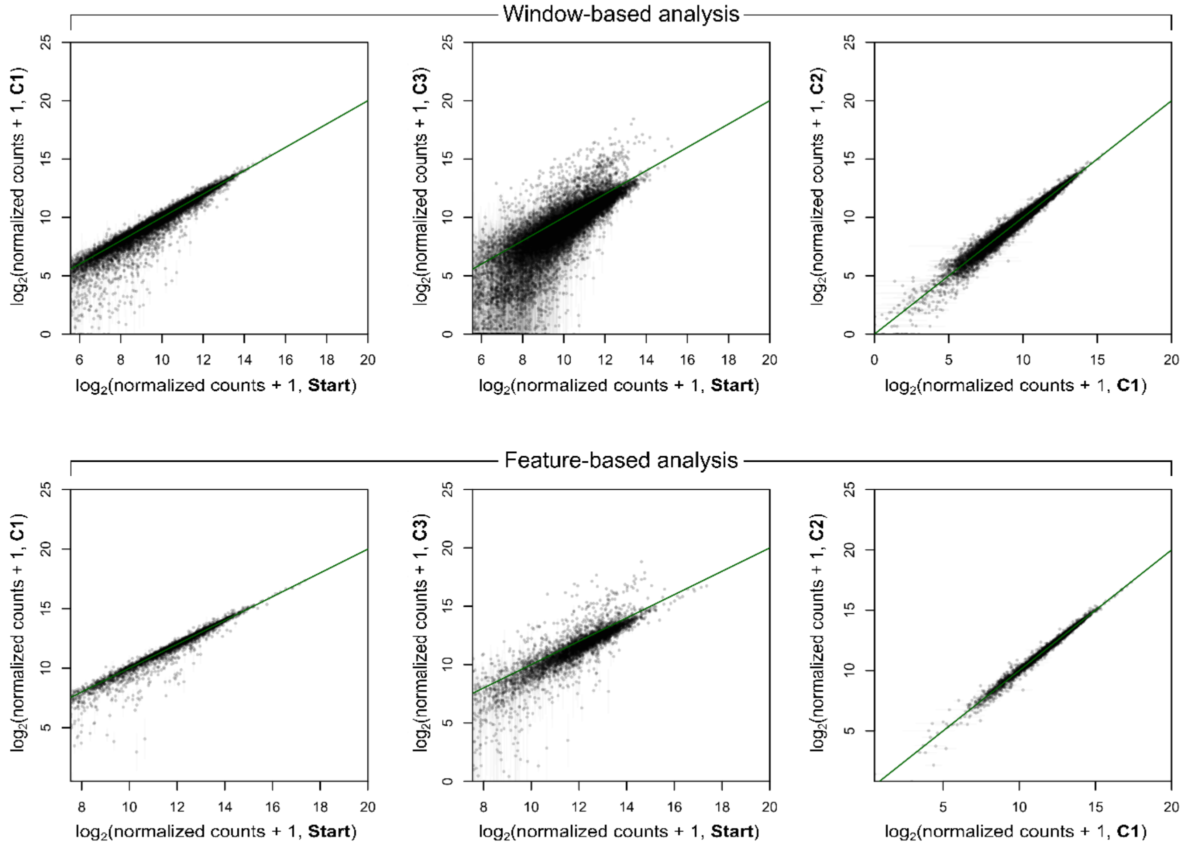

Figure S5. Comparison mutant abundances between controls and starting population. From left to right, comparison between starting population and C1 (left) and C3 (middle) controls, and comparison between C1 and C2 controls (right). Upper graphs show window-based analysis and lower graphs show feature-based analysis. C1 and C2 show relative mutant counts after growth on recovery plates in the absence and presence of *D. discoideum* respectively. C3 shows relative mutant counts for nutrient-deplete control condition (see Methods). Recovery plates have little to no effect on mutant abundances. Starvation causes depletion of certain mutants, but this depletion is minimal compared to the high predation conditions (Fig. 2).

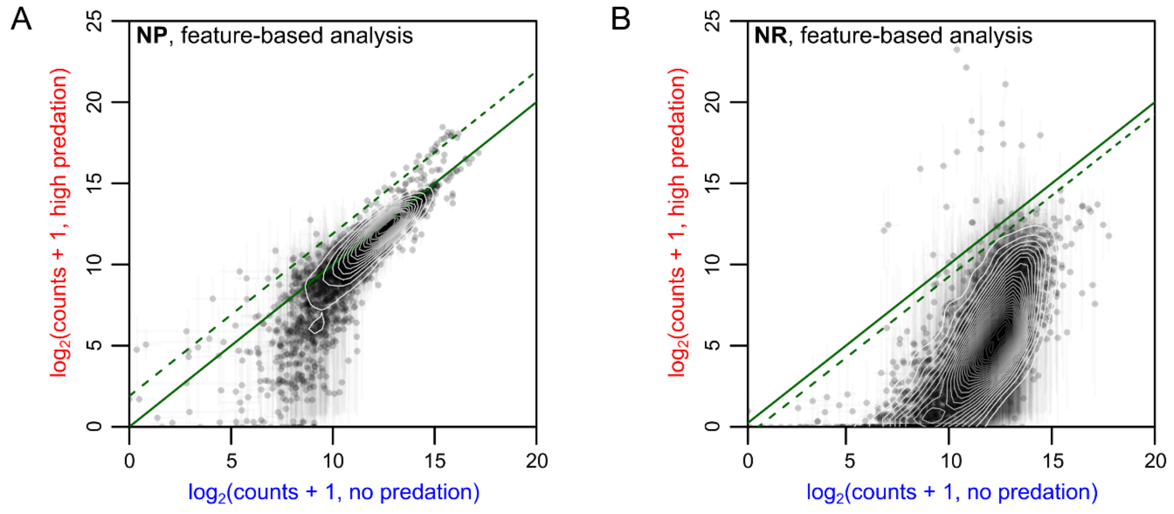

Figure S6. Feature-based comparison between mutant abundances in absence and presence of predation. Comparison of mutant abundances in absence (blue) and presence (red) of predation in (A) nutrient-poor suspension condition (NP) and (B) nutrient-rich surface condition (NR). See Fig. 2 for comparison to window-based analysis. All data is provided in Data S1.

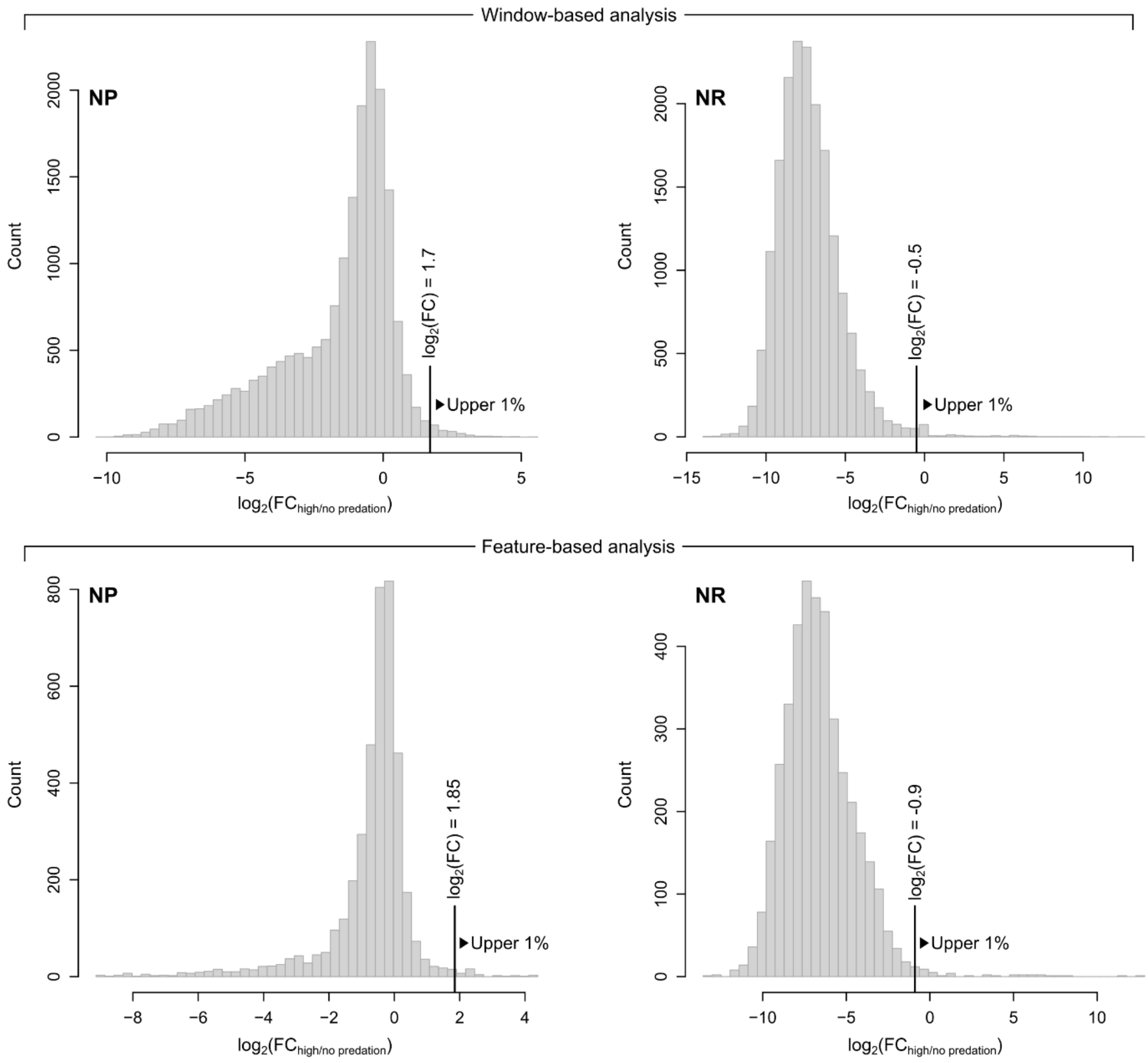

Figure S7. Log-fold changes in mutant abundances between no and high predation conditions in NP and NR samples. Top, log-fold changes between mutant abundance in no and high predation conditions in window-based analysis for NP and NR conditions. Bottom, log-fold changes between mutant abundance in no and high predation conditions in feature-based analysis for NP and NR conditions. Vertical lines show demarcation for upper 1% log-fold changes.

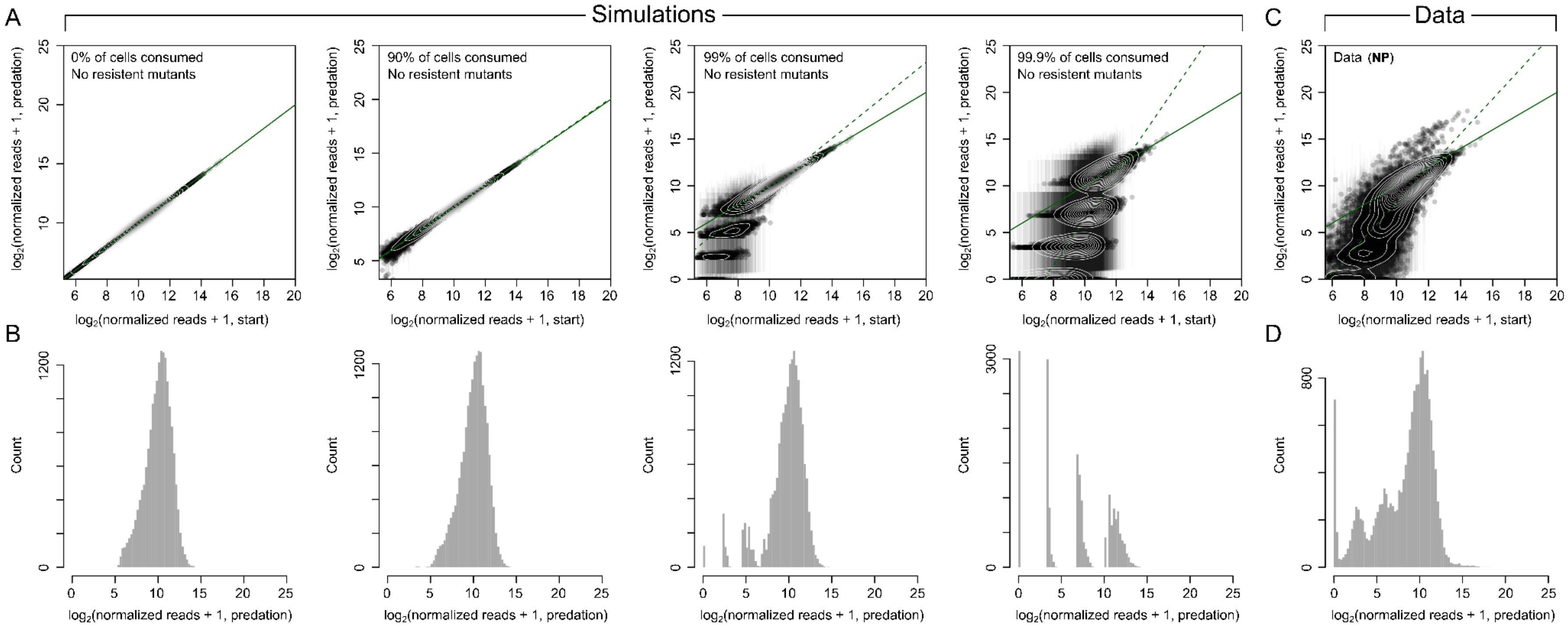

Figure S8. Comparison between simulated mutant distributions and real mutant distributions for NP growth condition. (A) Simulating expected mutant abundances after predation when 0%, 90%, 99%, and 99.9% of wildtypes are consumed, assuming that none of the transposon mutants is resistant. (B) Distribution of mutant abundances after predation in simulated data. (C) Mutant abundances after predation in NP growth condition, indicating that between 99% and 99.9% of susceptible cells are consumed. Thus, only 0.1% to 1% of non-resistant cells survive predation in the NP growth condition. (D) Distribution of mutant abundances after predation in NP growth condition. See Text S1 for further details. Solid green line shows diagonal and dashed green line shows regression.

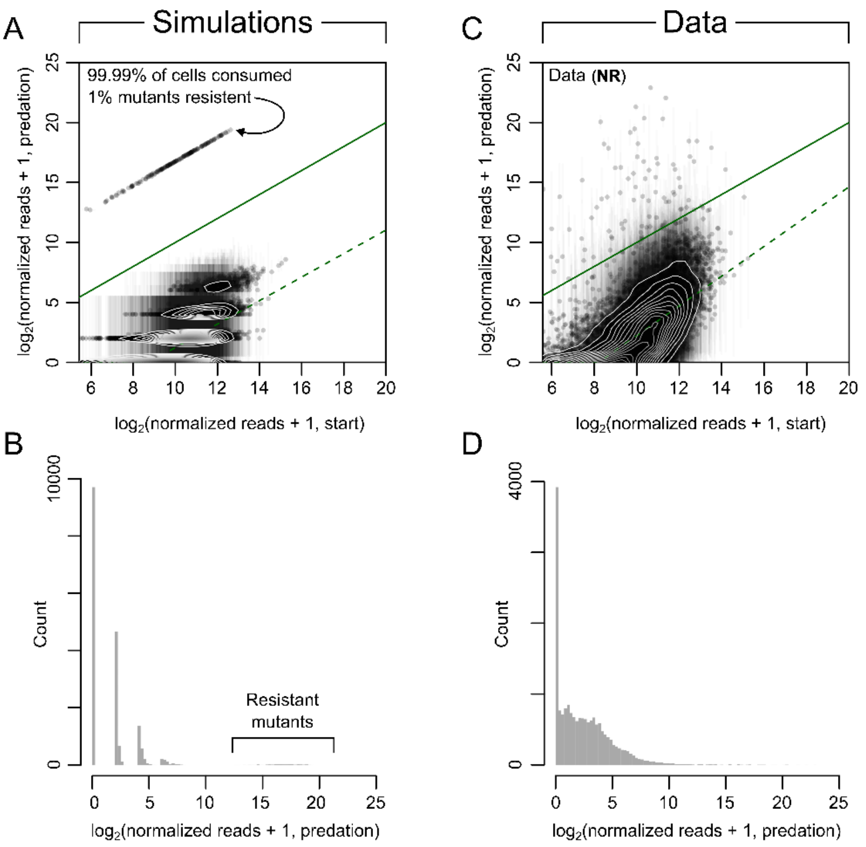

Figure S9. Comparison between simulated mutant distributions and real mutant distributions for NR growth condition. Based on direct measurements, an estimated fraction of 0.01% of non-resistant cells survive predation. In other words, 99.99% of the cells get consumed. (A) Simulation of expected mutant abundances after predation with a 99.99% consumption rate and a fraction of 1% of resistant transposon mutants. (B) Distribution of mutant abundances after predation in simulated data. (C) Mutant abundances after predation in NR growth condition. (D) Distribution of mutant abundances after predation in NR growth condition. See Text S1 for further details. Solid green lines show diagonal and dashed green line shows regression.

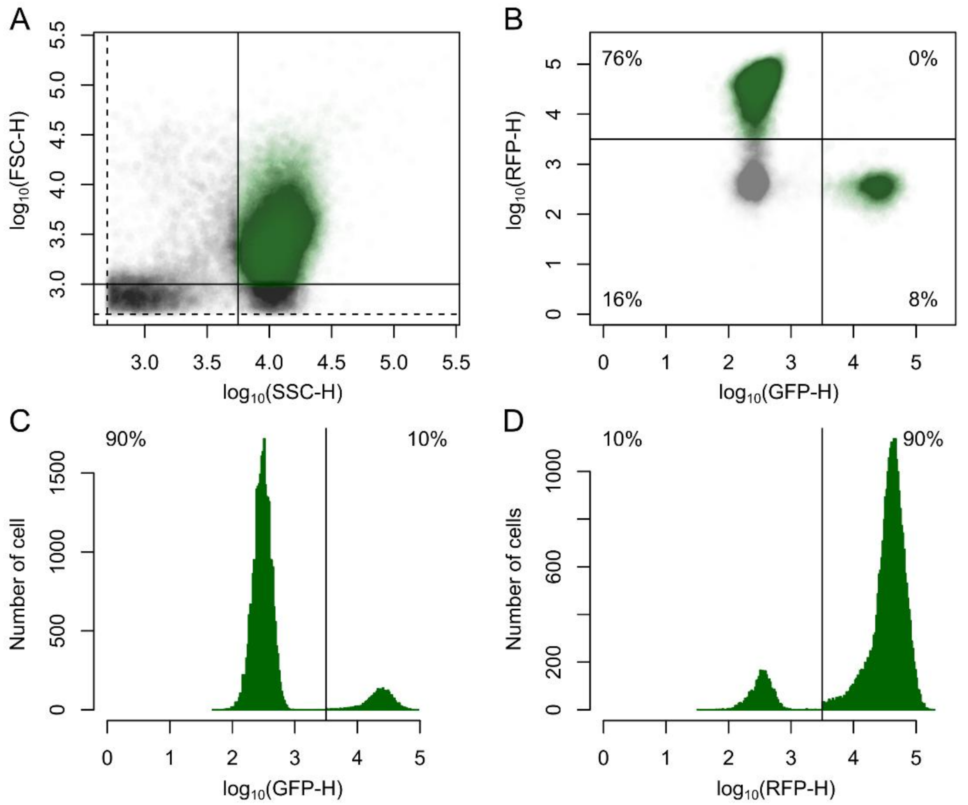

Figure S10. Example of flow cytometry results to count wildtype and mutant abundances in competition assays. (A) Forward scatter (FSC-H) and side-scatter (SSC-H) used to gate cells. (B) Percentage of cells that are RFP and GFP positive, corresponding to wildtype and resistant mutant cells respectively. (C) and (D) quantification of the fraction of GFP positive (10%) and RFP positive (90%) cells. For quantification in (D) non-fluorescent debris outside region of interest in (A) and (B) is ignored.

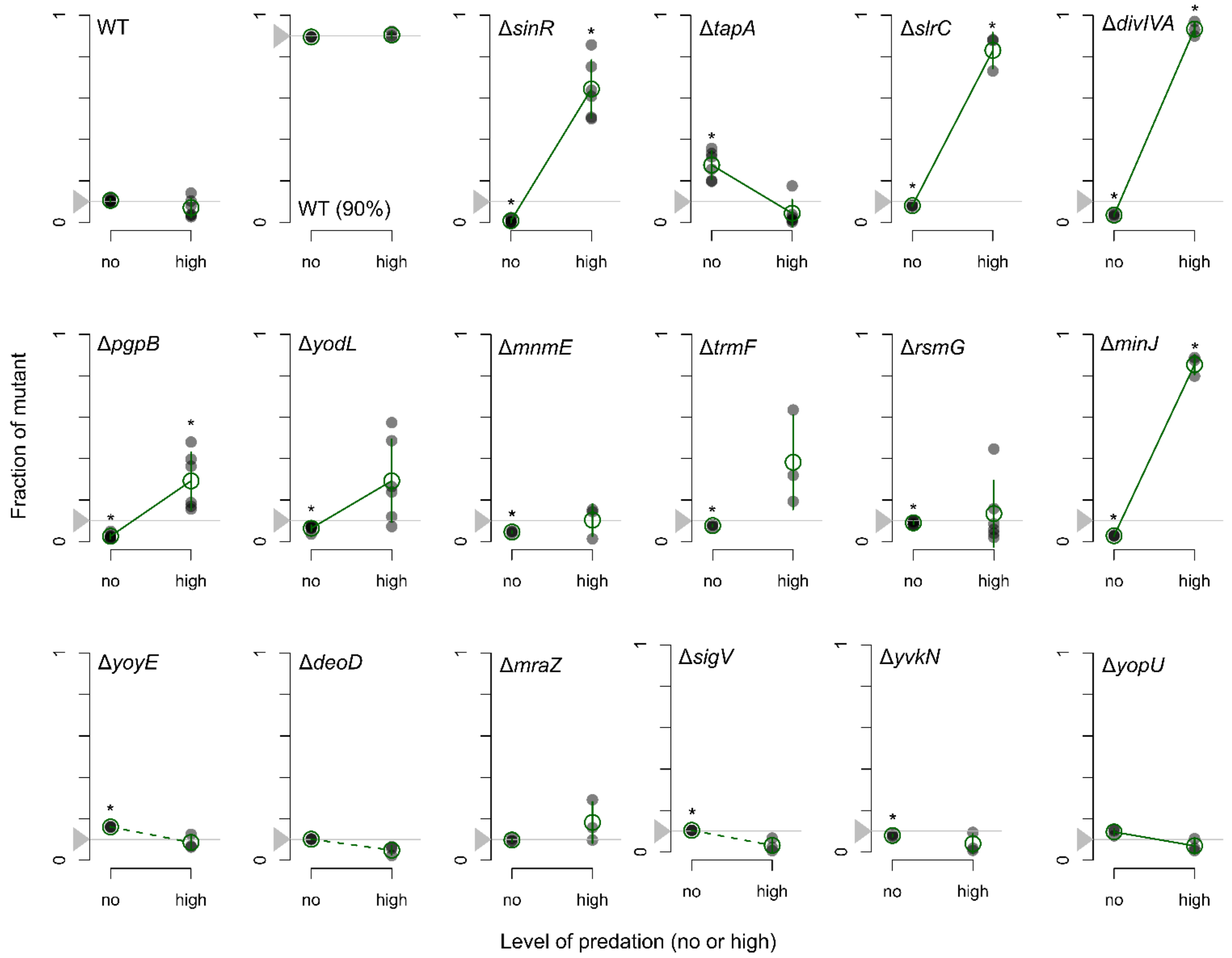

Figure S11. Competition assays between wildtype and mutant strains, using clean deletion. Following Fig. 3A, competition assays between wildtype and mutant strains in presence and absence of predation. Each knockout mutation is a clean deletion where the antibiotic cassette was removed (see Methods), thereby removing potential polar effects of antibiotic cassette on gene expression (Fig. 3B). When mutant frequencies significantly differ between no- and high-predation conditions regression lines (green) are shown. Dashed lines show trend ($0.05 < p < 0.1$). Asterisk show mutant frequencies that significantly deviate from starting frequency ($p < 0.05$, one-sample t-test, $mu = 0.1$). Most competition assays have the same qualitative outcome, with some exceptions. For example, the *tapA* knockout mutant is adaptive under predation with antibiotic cassette but selected against without; suggesting that the downstream overexpression of *tasA* is critical for the selective benefit (see Text S2). This suggests that overexpression of downstream genes might underlie fitness effect in transposon screen.

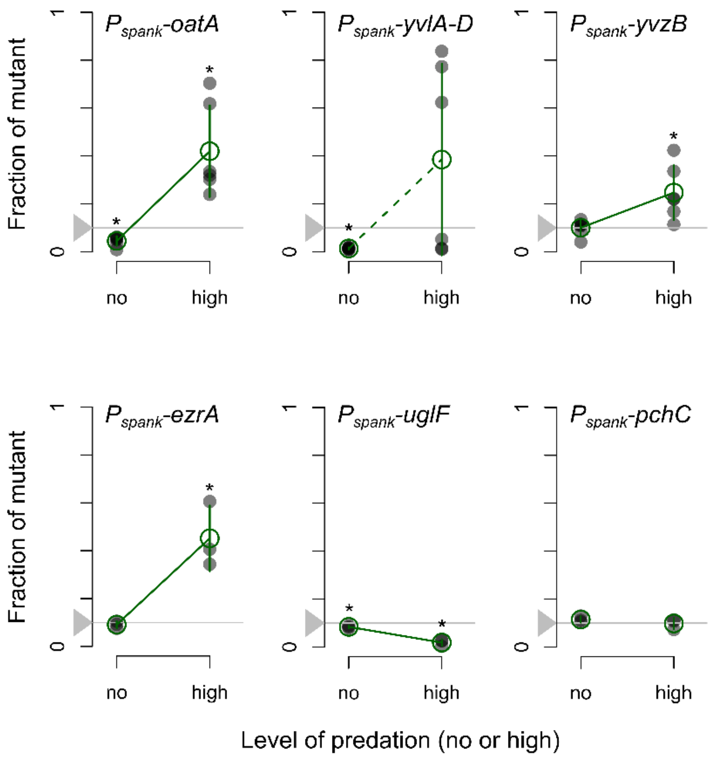

Figure S12. Competition assays between wildtype and overexpression mutant strains. Competition between wildtype and overexpression mutants, targeting genes and operons that based on the transposon screen could potentially mediate resistance when overexpressed. Genes are expressed under IPTG-inducible promoter with maximum induction (1mM IPTG; see Methods). Dashed line shows trend ($0.05 < p < 0.1$). For details see caption of Fig. S11.

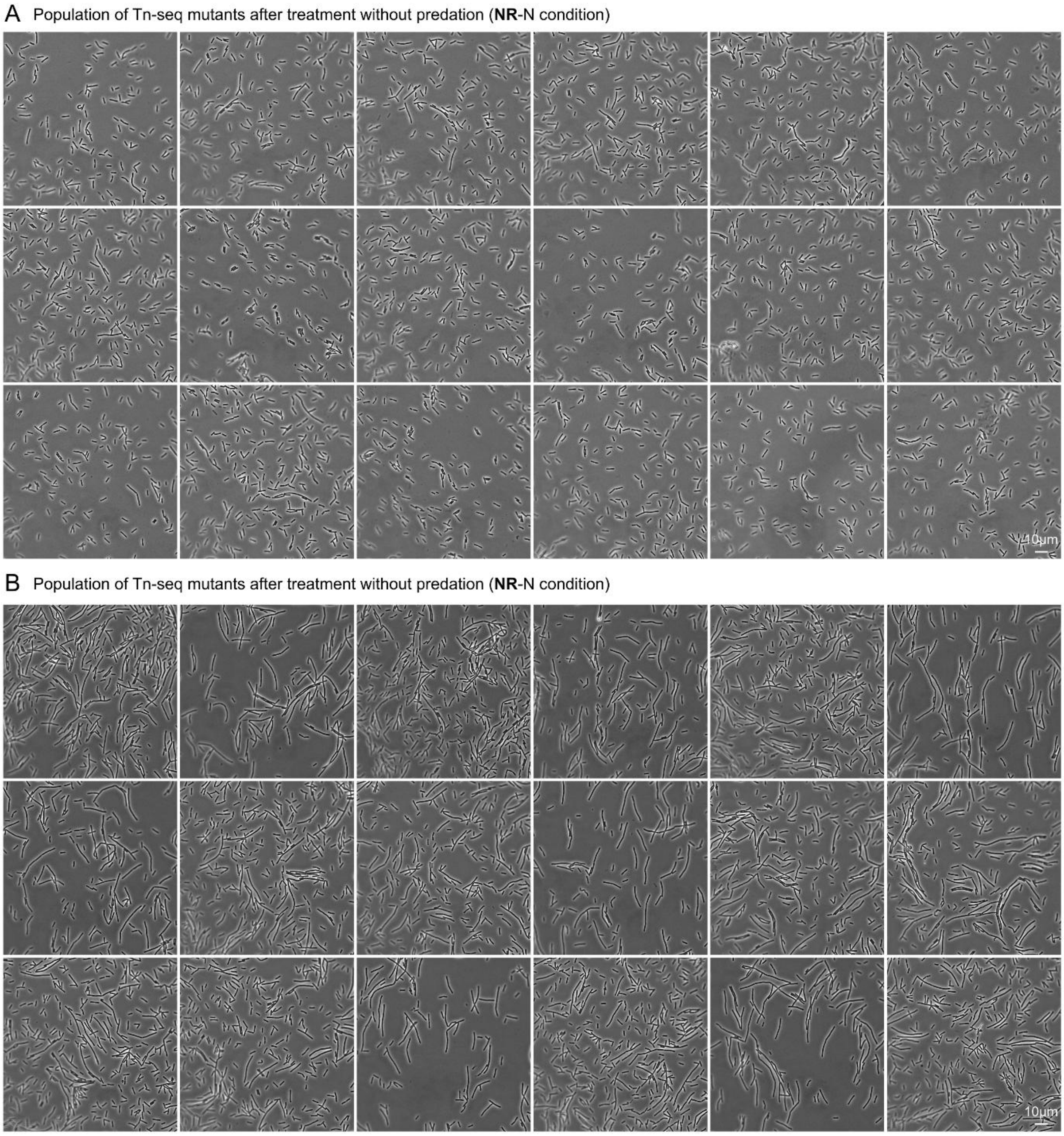

Figure S13. Population of transposon mutants after no and high predation treatment. Microscopy images of population of transposon mutants after transposon screen for the NR-N and NR-H conditions. Images were used for quantifications in Fig. 4B-C. Scale bar is 10μm.

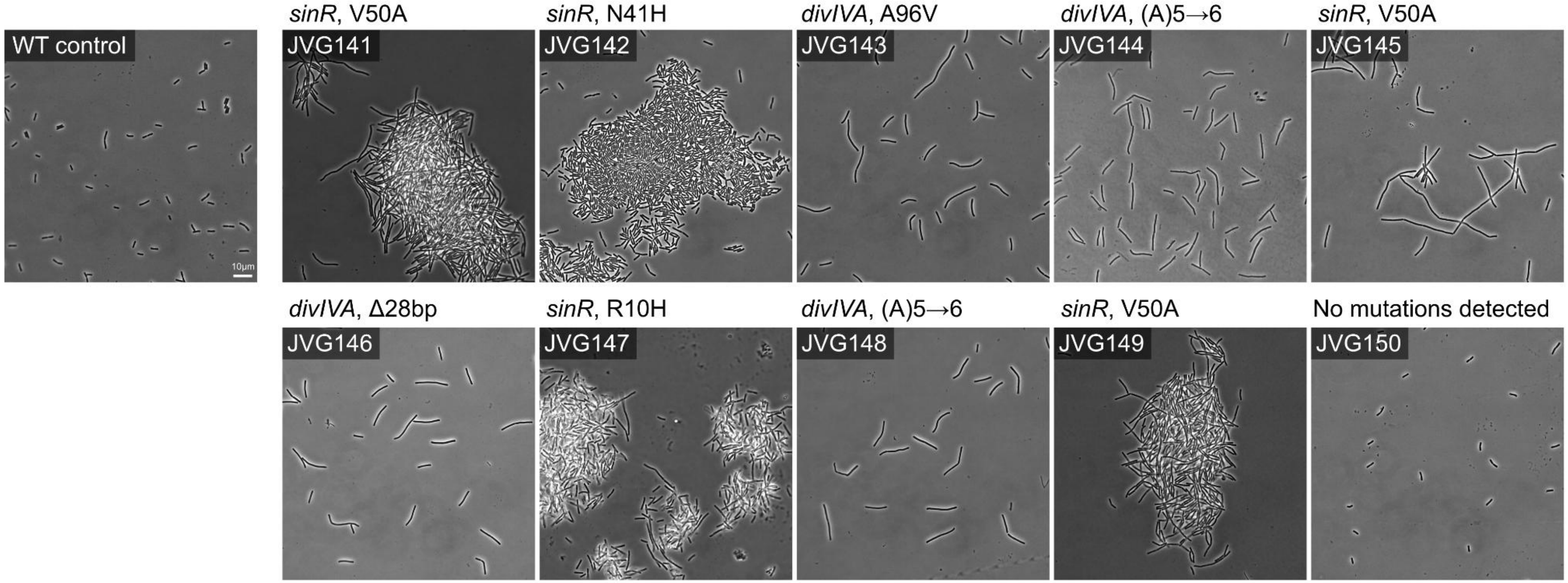

Figure S14. Resistant *B. subtilis* 168 isolates (JVG141-150) after exposure to predation, most of which have spontaneous mutations mediating defense. Microscopy images of stationary stage cultures of resistant colonies that emerged *de novo* with spontaneous mutants in both *sinR* and *divIVA* (see Fig. 5A). See Table S14 for details on exact mutations. Mutants show expected phenotypes: *sinR* mutations mostly result in aggregation and *divIVA* mutants in filamentation and elongated cell growth. In one of the resistant colonies no mutants were detected (JVG150) and indeed no microscopic phenotype could be detected either. Scale bar is 10μm.

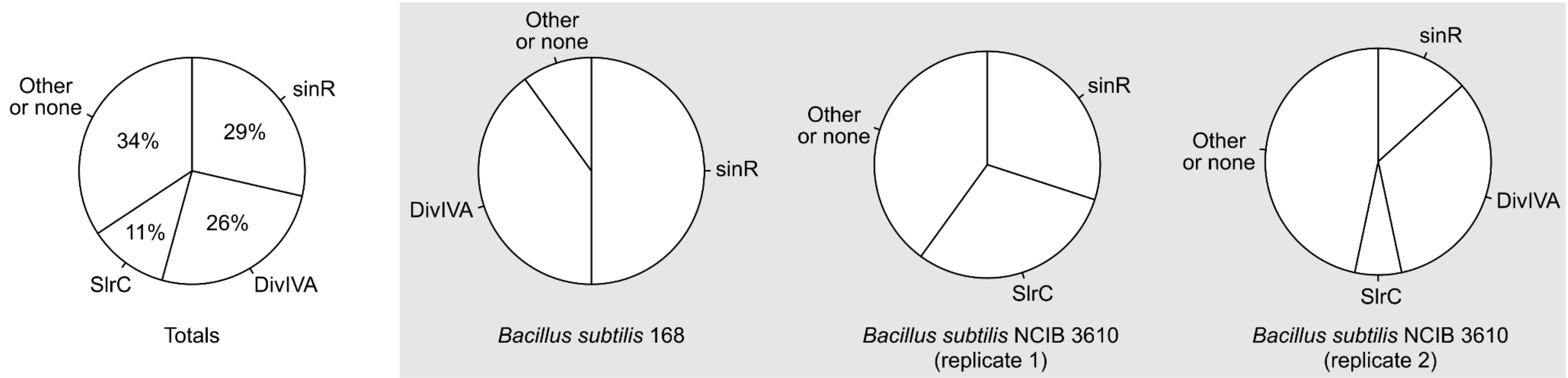

Figure S15. Mutations detected among resistant *B. subtilis* colonies. Left, mutations detected among all sequenced colonies (n=35), including colonies from *B. subtilis* 168 and *B. subtilis* NCIB 3610. Right, mutations detected from each of the three replicate experiments: one agar plate with *B. subtilis* 168 colonies and two independent agar plates with *B. subtilis* NCIB 3610 colonies. See Table S14, S15 and S16 for details.

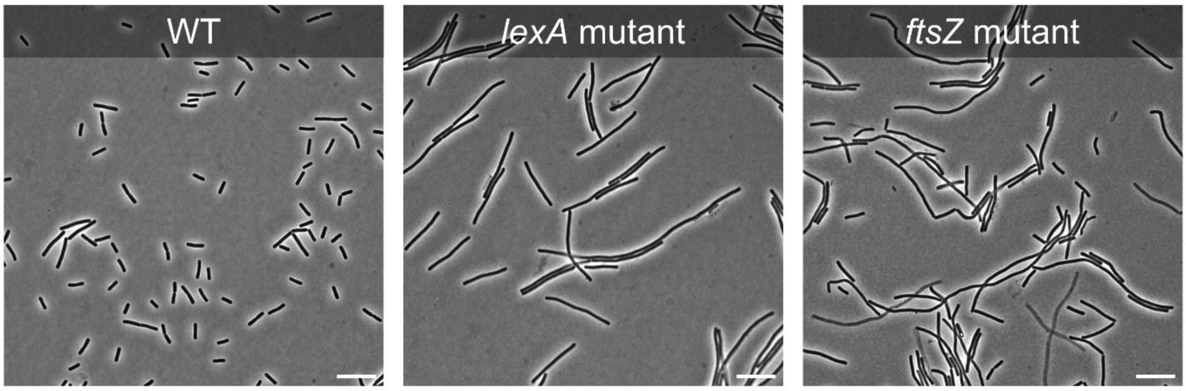

Figure S16. Microscopy images of wildtype and spontaneous *lexA* and *ftsZ* mutants. Microscopy images of wildtype *B. subtilis* NCIB 3610 and spontaneous mutants in *lexA* (JVG157; Δ1170bp) and *ftsZ* (JVG161; L249F and A278V). See Table S15 for details. For both resistant mutants there is strongly filamentous growth. Scale bar is 10μm.

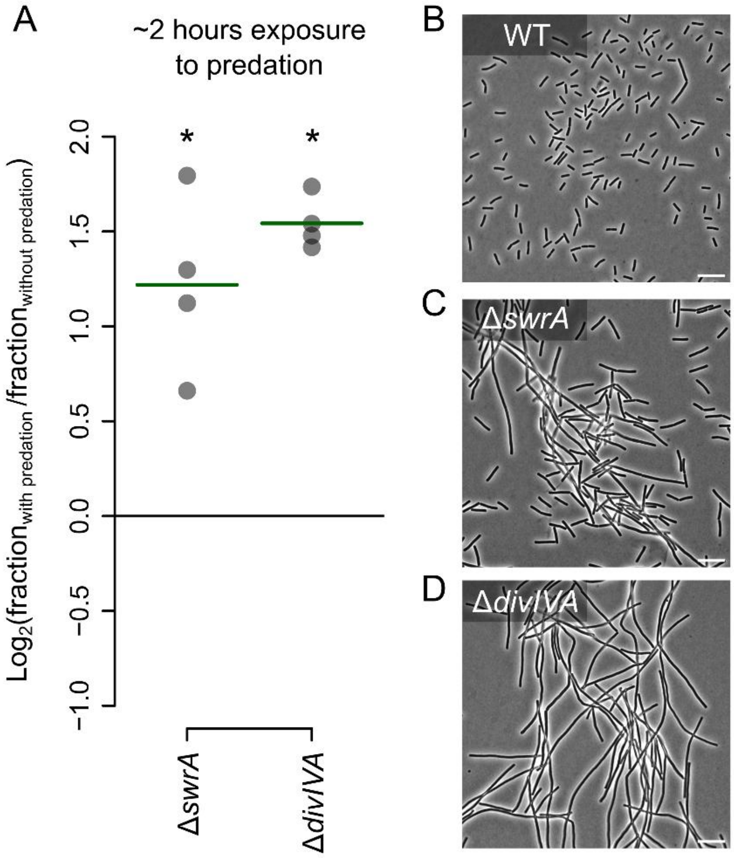

Figure S17. Competition assays between *B. subtilis* NCIB 3610 wildtype, Δ*swrA* and Δ*divIVA* mutants under predation. (A) Selective benefit of *B. subtilis* NCIB 3610 Δ*swrA* mutant and Δ*divIVA* (positive control) in the presence of predation in suspension condition (NP). Mutant (*gfp* expressing) and wildtype (*rfp* expressing) cells were mixed in suspension in a ratio of 10:90 and exposed to predation by *D. discoideum* AX2 for 2 to 2.5 hours. Fraction of mutants in the absence and presence of predation was determined through flow cytometry (e.g., Fig. S10). The selective benefit was measured by the relative enrichment of mutants in the presence and absence of predation. Horizontal line indicates no enrichment. Asterisk show significant enrichment of mutants under predation ($p < 0.05$, one-sample t-test, $mu = 0$). Mutants were moderately enriched, corresponding to the moderate enrichment of transposon mutants observed in the NP condition of the transposon screen (Fig. 2B). (B), (C) and (D) show microscopy images of wildtype, Δ*swrA* and Δ*divIVA* respectively. Scale bar is 10μm.

All supplementary data files (Data S1-S4, Movies S1-S12) are available through Zenodo (https://doi.org/10.5281/zenodo.14871691).

Movie S1. Predation of *B. subtilis* 168 wildtype cells (JVG319) by *D. discoideum*. Scale bar is 10µm.

Movie S2. Predation of *B. subtilis* 168 Δ*sinR* (JVG332) by *D. discoideum*. Scale bar is 10µm.

Movie S3. Predation of *B. subtilis* 168 Δ*slrC* (JVG327) by *D. discoideum*. Scale bar is 10µm.

Movie S4. Predation of *B. subtilis* 168 Δ*divIVA* (JVG336) by *D. discoideum*. Scale bar is 10µm.

Movie S5. Predation of spontaneous *sinR* mutant (JVG103) by *D. discoideum*. Scale bar is 10µm.

Movie S6. Predation of spontaneous *lexA* mutant (JVG157) by *D. discoideum*. Scale bar is 10µm.

Movie S7. Predation of *B. subtilis* NCIB 3610 Δ*swrA* (BSR1031) by *D. discoideum*. Scale bar is 10µm.

Movie S8. Predation of *B. subtilis* NCIB 3610 Δ*swrA* (BSR1031) by *D. discoideum*. Scale bar is 10µm.

Movie S9. Predation of *B. subtilis* NCIB 3610 Δ*swrA* (BSR1031) by *D. discoideum*. Scale bar is 10µm.

Movie S10. Predation of *B. subtilis* NCIB 3610 Δ*divIVA* (VSG131) by *D. discoideum*. Scale bar is 10µm.

Movie S11. Predation of *B. subtilis* NCIB 3610 Δ*divIVA* (VSG131) by *D. discoideum*. Scale bar is 10µm.

Movie S12. Predation of *B. subtilis* NCIB 3610 Δ*divIVA* (VSG131) by *D. discoideum*. Scale bar is 10µm.

Table S1. Material list

n	Description	Supplier, product number
1	10μL inoculation loop	BRAND, BR452210
2	L-shaped spreader	Fisher, 14665230
3	Bulldog Disposable Hemocytometer, Neubauer Improved	Fisher, NC0435502
4	Bacteriological Petri Dish (100mmx15mm)	Falcon, 35109
5	24-well Multiwell Flat Bottom Cell Culture plate	Falcon, 353226
6	Petri-dishes	Corning, Falcon, 351029
7	15 mL Conical Centrifuge Tubes	Falcon, 352097
8	Kanamycin sulfate	Sigma-Aldrich, K1377
9	Chloramphenicol	Sigma-Aldrich, C0378
10	Erythromycin	Sigma-Aldrich, E5389
11	Lincomycin hydrochloride	Sigma-Aldrich, L2774
12	Spectinomycin dihydrochloride pentahydrate	Sigma-Aldrich, S9007
13	Ampicillin sodium salt	Sigma-Aldrich, A9518
14	Lysozyme (from chicken egg white)	Sigma-Aldrich, L6876-10g
15	MmeI restriction enzyme	New England Biolabs, R0637L
16	T4 DNA ligase	New England Biolabs, M0202L
17	10x T4 DNA ligase buffer	New England Biolabs, B0202S
18	Quick CIP	New England Biolabs, M0525S
19	Q5 Hot Start High-Fidelity DNA Polymerase	New England Biolabs, M0493L
20	5x Q5 buffer	New England Biolabs, B0927S
21	10mM dNTP	New England Biolabs, N0447S
22	6x Loading buffer	New England Biolabs, B7024S
23	Zymo DNA Clean & Concentrator-5 Kit	Zymo Research, D4004
24	Wizard Genomic DNA Purification Kit	Promega, A1120
25	EB buffer	Qiagen, 19086
26	DNeasy Blood & Tissue Kit	Qiagen, 69506
27	QIAquick PCR purification kit	Qiagen, 28104
28	MinElute Gel Extraction Kit	Qiagen, 28604
29	Agilent High Sensitivity DNA Kit	Agilent, UFUCOP-5067-4626
30	Novex TBE Gel, 8%, 12-well	Invitrogen, EC62152BOX
31	0.5mL DNA LoBind Tube	Eppendorf, 0030 108.35
32	1.5mL DNA LoBind Tube	Eppendorf, 0030 108.051
33	SYBR Gold Nucleic Acid Gel Stain	Invitrogen, S11494
34	Ultra-Low Range DNA Ladder	Invitrogen, 10597012
35	NEBuilder HiFi DNA Assembly Cloning Kit	New England Biolabs, E5520S
36	EcoRV-HF restriction enzyme	New England Biolabs, R3195L
37	BamHI-HF restriction enzyme	New England Biolabs, R3136L
38	ScaI-HF restriction enzyme	New England Biolabs, R3122L

Table S2. LPB plates

n	W	Product	Supplier
1	1g	Lactose (alpha-lactose monohydrate)	Sigma-Aldrich, L3625-1kg
2	1g	Bacto peptone	Difco, 0118-17-10
3	5.1g	Sodium phosphate dibasic heptahydrate ($Na_2HPO_4{\cdot}7H_2O$)	Fisher, S373-3
4	4.2g	Potassium phosphate monobasic (KH_2PO_4)	Sigma-Aldrich, P0662-1kg
5	20g	Agar	BD, 214030
		Add 1L dH_2O	

Table S3. Custom SM medium

n	W	Product	Supplier
1	10g	D(+)glucose ($C_6H_{12}O_6$)	Sigma-Aldrich, D9434-1kg
2	10g	Bacto Peptone	BD Bacto Peptone REF211677
3	1g	Yeast extract	BD Bacto Yeast Extract REF212750
4	1g	Magnesium sulfate heptahydrate ($MgSO_4{\cdot}7H_2O$)	Fisher, M63-500
5	1.9g	Potassium phosphate monobasic (KH_2PO_4)	Sigma-Aldrich P0662-1kg
6	0.6g	Potassium phosphate dibasic (K_2HPO_4)	Sigma-Aldrich, P8281-500g
7	20g	Agar	BD, 214030
		dH_2O to 1L	

Table S4. Custom SM/5 medium

n	W	Product	Supplier
1	2g	D(+)glucose ($C_6H_{12}O_6$)	Sigma-Aldrich, D9434-1kg
2	2g	Bacto Peptone	BD Bacto Peptone REF211677
3	0.2g	Yeast extract	BD Bacto Yeast Extract REF212750
4	1g	Magnesium sulfate heptahydrate (MgSO4·7H2O)	Fisher, M63-500
5	1.9g	Potassium phosphate monobasic (KH_2PO_4)	Sigma-Aldrich, P0662-1kg
6	0.6g	Potassium phosphate dibasic (K_2HPO_4)	Sigma-Aldrich, P8281-500g
7	20g	Agar	BD, 214030
		dH_2O to 1L	

Table S5. 10x KK2 stock solution*

n	W	Product	Supplier
1	22g	Potassium phosphate monobasic (KH_2PO_4)	Sigma-Aldrich, P0662-500G
2	7g	Potassium phosphate dibasic (K_2HPO_4)	Sigma-Aldrich, P8281-500G
		dH_2O to 1L	

*For 1x KK2-MC, the buffer is supplemented with magnesium chloride and calcium chloride to a final concentration of 50uM $MgCl_2$ (Sigma, M9272-1KG) and 50uM $CaCl_2$ (Sigma, C3881-500G).

Table S6. 10x SorMC stock solution*

n	W	Product	Supplier
1	20.41g	Potassium phosphate monobasic (KH_2PO_4)	Sigma-Aldrich, P0662-500G
2	3.56g	Sodium phosphate dibasic dihydrate ($Na_2HPO_4 \cdot 2H_2O$)	Sigma-Aldrich, 71643-250G
		dH_2O to 1L	

* For final 1x SorMC, buffer is supplemented with magnesium chloride and calcium chloride to a final concentration of 50uM $MgCl_2$ (Sigma, M9272-1KG) and 50uM $CaCl_2$ (Sigma, C3881-500G).

Table S7. LB or LB agar

n	W	Product	Supplier
1	20g	LB broth (Lennox)	Fisher, BP1427-2
2	18g	**Optional:** Agar	BD, 214030
		dH_2O to 1L	

Table S8. Oligos

Name	Description	Oligo
		**Tn-library prep and sequencing primers (see also Figure S1)**
oCJ26	Adapter upper	5′ P-GTGTGATCGT CGGACTGTAG AACTCTGAAC CTGTC-P 3′
oCJ25	Adapter lower	5′ GTTCAGAGTT CTACAGTCCG ACGATCACAC NN 3′
ocJ22-ind	p7 primer [i7] index	5′ CAAGCAGAAG ACGGCATACG AGAT**XXXXXX** TGTGTGAGAC CGGGGACTTA TCATCCAACC TGT 3′
ocJ23-ind	p5 primer [i5] index	5′ AATGATACGG CGACCACCGA GATCTACAC**X XXXXX**GACAG GTTCAGAGTT CTACAGTCCGA 3′
oCJ24	Sequencing primer	5′ GACAGGTTCA GAGTTCTACA GTCCGACGAT CACAC 3′
oCJ27	[i7] index primer	5′ ACAGGTTGGA TGATAAGTCC CCGGTCTCAC ACA 3′
NA	[i5] index primer	5′ GTGTGATCGT CGGACTGTAG AACTCTGAAC CTGTC 3′
		**Cloning oligos**
P_75.F	*spcR* forward	5′ GACCATGCAGAGGATGATGCTCGTGACGGT CGTAACGTGACTGGCAAGAG 3′
P_75.R	*spcR* reverse	5′ CAACTGGTAATGGTAGCGACCGGCGCTCAG GAGATCCCCCTATGCAAGGG 3′
P_77.F	*cmR* forward	5′ GACCATGCAGAGGATGATGCTCGTGACGGT TGACAGCTTATCATCGGCAA 3′
P_77.R	*cmR* reverse	5′ CAACTGGTAATGGTAGCGACCGGCGCTCAG CACCATACCCACGCCGAA 3′
P_69.F	*yvlABCD* forward	5′ GGCTAC GTCGAC TCACAATAGGAGTGTGGAATGA 3′
P_69.R	*yvlABCD* reverse	5′ GGCTAC GCATGC CAACAGAGGGAGTGCAATCG 3′
P_70.F	*yvzB* forward	5′ GGCTAC GTCGAC GATCTTGGTGCAATTAAAGATG 3′
P_70.R	*yvzB* reverse	5′ GGCTAC GCATGC TCAGAAAGAGATAGCCGTTCAA 3′
P_119.F	*ezrA* forward	5′ GGCTAC GTCGAC GATTAACTAATAAGGAGGACAAAC ATGGAGTTTGTCATTGGATTATTAATTGTACTG
P_71.R	*ezrA* reverse	5′ GGCTAC GCTAGC GTGCTTCCAGGAATCGTCG 3′
P_120.F	*uglF* forward	5′ GGCTAC GTCGAC GATTAACTAATAAGGAGGACAAAC ATGAATATAACAGTCATCGGAACA 3′
P_72.R	*uglF* reverse	5′ GGCTAC GCATGC CCAGCCTTTCTTTTTCATCT 3′
P_121.F	*pchC* forward	5′ GGCTAC GTCGAC GATTAACTAATAAGGAGGACAAAC ATGACCGGAATGGTAACG 3′
P_73.R	*pchC* reveres	5′ GGCTAC GCATGC GATTGGCTCATGTTTTACTC 3′
P_74.F	*oatA* forward	5′ GGCTAC GTCGAC TACTATTGGAGGAATACCCA 3′
P_74.R	*oatA* reverse	5′ GGCTAC GCATGC GTGATCTGTATAGGATCTCT 3′

Table S9. Strain list

n	Identifier	Strain	Genotype	Parent strain*
1	JVG65	*Dictyostelium discoideum* NC4	Kenneth Raper	dictyBase DBS0304666
2	JVG68	*Dictyostelium discoideum* AX2	Rob Kay	dictyBase DBS0235521
3	MG038***	*Dictyostelium discoideum* AX2	Δact5::mCherry [hygR]	dictyBase DBS0235521
4	JVG72	*Escherichia coli* B/r	Ikuo Takeuchi / John Bonner	dictyBase DBS0305924
5	JVG31	*Escherichia coli* K-12	Δ(araD-araB)567, ΔlacZ4787(::rrnB-3), Δ(phoB-phoR)580, λ-, galU95, ΔuidA3::pir+, recA1, endA9(del-ins)::FRT, rph-1, Δ(rhaD-rhaB)568, hsdR514	BW25141
6	JVG1	*Bacillus subtilis* NCIB 3610	Wildtype	CAG74361
7	JVG12	*Bacillus subtilis* 168	Wildtype	BGSC; 1A1
8	JVG319	*Bacillus subtilis* 168	sacA::Pveg-eGFP [spcR]	JVG12
9	JVG320	*Bacillus subtilis* 168	sacA::Pveg-mRFP1 [spcR]	JVG12
10	JVG340	*Bacillus subtilis* 168	ΔpgpB::lox-kan-lox, sacA::Pveg-eGFP [spcR]	BKK19650
11	JVG370	*Bacillus subtilis* 168	ΔpgpB, sacA::Pveg-eGFP [spcR]	BKK19650
12	JVG339	*Bacillus subtilis* 168	ΔyodL::lox-kan-lox, sacA::Pveg-eGFP [spcR]	BKK19640
13	JVG369	*Bacillus subtilis* 168	ΔyodL, sacA::Pveg-eGFP [spcR]	BKK19640
14	JVG338	*Bacillus subtilis* 168	ΔyoyE::lox-kan-lox, sacA::Pveg-eGFP [spcR]	BKK19639
15	JVG368	*Bacillus subtilis* 168	ΔyoyE, sacA::Pveg-eGFP [spcR]	BKK19639
16	JVG337	*Bacillus subtilis* 168	ΔdeoD::lox-kan-lox, sacA::Pveg-eGFP [spcR]	BKK19630
17	JVG367	*Bacillus subtilis* 168	ΔdeoD, sacA::Pveg-eGFP [spcR]	BKK19630
18	JVG336	*Bacillus subtilis* 168	ΔdivIVA::lox-kan-lox, sacA::Pveg-eGFP [spcR]	BKK15420
19	JVG366	*Bacillus subtilis* 168	ΔdivIVA, sacA::Pveg-eGFP [spcR]	BKK15420
20	JVG399	*Bacillus subtilis* 168	ΔezrA, sacA::Pveg-eGFP [cmR], amyE::Pspac-ezrA [spcR]	BKK29610
21	JVG400	*Bacillus subtilis* 168	ΔezrA, sacA::Pveg-eGFP [cmR], amyE::Pspac_low-ezrA [spcR]	BKK29610
22	JVG458	*Bacillus subtilis* 168	sacA::Pveg-eGFP [cmR], amyE::Pspac-RBS[IPTG]-ezrA[spcR]	JVG12
23	JVG459	*Bacillus subtilis* 168	sacA::Pveg-eGFP [cmR], amyE::Pspac_low-RBS[IPTG]-ezrA [spcR]	JVG12
24	JVG349	*Bacillus subtilis* 168	ΔminJ::lox-kan-lox, sacA::Pveg-eGFP [spcR]	BKK35220
25	JVG379	*Bacillus subtilis* 168	ΔminJ, sacA::Pveg-eGFP [spcR]	BKK35220
26	JVG354	*Bacillus subtilis* 168	ΔmnmE::lox-kan-lox, sacA::Pveg-eGFP [spcR]	BKK41020
27	JVG384	*Bacillus subtilis* 168	ΔmnmE, sacA::Pveg-eGFP [spcR]	BKK41020
28	JVG353	*Bacillus subtilis* 168	ΔtrmF::lox-kan-lox, sacA::Pveg-eGFP [spcR]	BKK41010
29	JVG383	*Bacillus subtilis* 168	ΔtrmF, sacA::Pveg-eGFP [spcR]	BKK41010
30	JVG352	*Bacillus subtilis* 168	ΔrsmG::lox-kan-lox, sacA::Pveg-eGFP [spcR]	BKK41000
31	JVG382	*Bacillus subtilis* 168	ΔrsmG, sacA::Pveg-eGFP [spcR]	BKK41000
32	JVG335	*Bacillus subtilis* 168	ΔmraZ::lox-kan-lox, sacA::Pveg-eGFP [spcR]	BKK15130
33	JVG365	*Bacillus subtilis* 168	ΔmraZ, sacA::Pveg-eGFP [spcR]	BKK15130
34	JVG343	*Bacillus subtilis* 168	ΔsigV::lox-kan-lox, sacA::Pveg-eGFP [spcR]	BKK27120
35	JVG373	*Bacillus subtilis* 168	ΔsigV, sacA::Pveg-eGFP [spcR]	BKK27120
36	JVG405	*Bacillus subtilis* 168	ΔoatA, sacA::Pveg-eGFP [cmR], amyE::Pspac-oatA [spcR]	BKK27140
37	JVG406	*Bacillus subtilis* 168	ΔoatA, sacA::Pveg-eGFP [cmR], amyE::Pspac_low-oatA [spcR]	BKK27140
38	JVG350	*Bacillus subtilis* 168	ΔptpZ::lox-kan-lox, sacA::Pveg-eGFP [spcR]	BKK36240
39	JVG380	*Bacillus subtilis* 168	ΔptpZ, sacA::Pveg-eGFP [spcR]	BKK36240
40	JVG401	*Bacillus subtilis* 168	ΔuglF, sacA::Pveg-eGFP [cmR], amyE::Pspac-uglF [spcR]	BKE36230
41	JVG402	*Bacillus subtilis* 168	ΔuglF, sacA::Pveg-eGFP [cmR], amyE::Pspac_low-uglF [spcR]	BKE36230
42	JVG460	*Bacillus subtilis* 168	sacA::Pveg-eGFP [cmR], amyE::Pspac-RBS[IPTG]-uglF [spcR]	JVG12
43	JVG461	*Bacillus subtilis* 168	sacA::Pveg-eGFP [cmR], amyE::Pspac_low-RBS[IPTG]-uglF [spcR]	JVG12
44	JVG403	*Bacillus subtilis* 168	ΔpchC, sacA::Pveg-eGFP [cmR], amyE::Pspac-pchC [spcR]	BKK35070
45	JVG404	*Bacillus subtilis* 168	ΔpchC, sacA::Pveg-eGFP [cmR], amyE::Pspac_low -pchC [spcR]	BKK35070
46	JVG462	*Bacillus subtilis* 168	sacA::Pveg-eGFP [cmR], amyE::Pspac-RBS[IPTG]-pchC [spcR]	JVG12
47	JVG463	*Bacillus subtilis* 168	sacA::Pveg-eGFP [cmR], amyE::Pspac_low-RBS[IPTG]-pchC [spcR]	JVG12
48	JVG347	*Bacillus subtilis* 168	ΔyvkN::lox-kan-lox, sacA::Pveg-eGFP [spcR]	BKK35140
49	JVG377	*Bacillus subtilis* 168	ΔyvkN, sacA::Pveg-eGFP [spcR]	BKK35140
50	JVG385	*Bacillus subtilis* 168	ΔyvlABCD::kan, sacA::Pveg-eGFP [cmR], amyE::Pspac-yvlABCD [spcR]	CAG74483
51	JVG386	*Bacillus subtilis* 168	ΔyvlABCD::kan, sacA::Pveg-eGFP [cmR], amyE::Pspac_low-yvlABCD [spcR]	CAG74483
52	JVG397	*Bacillus subtilis* 168	ΔyvzB, sacA::Pveg-eGFP [cmR], amyE::Pspac-yvzB [spcR]	BKK35150
53	JVG398	*Bacillus subtilis* 168	ΔyvzB, sacA::Pveg-eGFP [cmR], amyE::Pspac_low-yvzB [spcR]	BKK35150
54	JVG341	*Bacillus subtilis* 168	ΔyopU::lox-kan-lox, sacA::Pveg-eGFP [spcR]	BKK20760
55	JVG371	*Bacillus subtilis* 168	ΔyopU, sacA::Pveg-eGFP [spcR]	BKK20760
56	JVG429	*Bacillus subtilis* 168	ΔslrA::lox-kan-lox, sacA::Pveg-eGFP [spcR]	BKK38229
57	JVG331	*Bacillus subtilis* 168	ΔsinI::lox-kan-lox, sacA::Pveg-eGFP [spcR]	CAG74351
58	JVG361	*Bacillus subtilis* 168	ΔsinI, sacA::Pveg-eGFP [spcR]	CAG74351
59	JVG332	*Bacillus subtilis* 168	ΔsinR::lox-kan-lox, sacA::Pveg-eGFP [spcR]	CAG74353
60	JVG362	*Bacillus subtilis* 168	ΔsinR, sacA::Pveg-eGFP [spcR]	CAG74353
61	JVG330	*Bacillus subtilis* 168	Δspo0A::lox-kan-lox, sacA::Pveg-eGFP [spcR]	CAG74349
62	JVG360	*Bacillus subtilis* 168	Δspo0A, sacA::Pveg-eGFP [spcR]	CAG74349

63	JVG342	*Bacillus subtilis* 168	ΔtapA::lox-kan-lox, sacA::Pveg-eGFP [spcR]	BKK24640
64	JVG372	*Bacillus subtilis* 168	ΔtapA, sacA::Pveg-eGFP [spcR]	BKK24640
65	JVG327	*Bacillus subtilis* 168	ΔywcC::lox-kan-lox, sacA::Pveg-eGFP [spcR]	BKK38220
66	JVG357	*Bacillus subtilis* 168	ΔywcC, sacA::Pveg-eGFP [spcR]	BKK38220
67	JVG432	*Bacillus subtilis* 168	ΔywcD::lox-kan-lox, sacA::Pveg-eGFP [cmR], amyE::Pspac-ywcC-ywcD [spcR]	BKK38210
68	JVG436	*Bacillus subtilis* 168	ΔywcD, sacA::Pveg-eGFP [cmR], amyE::Pspac-ywcC-ywcD [spcR]	BKK38210
69	JVG466	*Bacillus subtilis* 168	sacA::Pveg-eGFP [cmR], amyE::Pspac-RBS[IPTG]-ywcC-ywcD [spcR]	JVG12
70	JVG467	*Bacillus subtilis* 168	sacA::Pveg-eGFP [cmR], amyE::Pspac_low-RBS[IPTG]-ywcC-ywcD [spcR]	JVG12
71	JVG351	*Bacillus subtilis* 168	ΔywcD::lox-kan-lox, sacA::Pveg-eGFP [spcR]	BKK38210
72	JVG381	*Bacillus subtilis* 168	ΔywcD, sacA::Pveg-eGFP [spcR]	BKK38210
73	JVG431	*Bacillus subtilis* 168	ΔywcD::lox-kan-lox, sacA::Pveg-eGFP [cmR], amyE::Pspac-ywcD [spcR]	BKK38210
74	JVG435	*Bacillus subtilis* 168	ΔywcD, sacA::Pveg-eGFP [cmR], amyE::Pspac-ywcD [spcR]	BKK38210
75	JVG464	*Bacillus subtilis* 168	sacA::Pveg-eGFP [cmR], amyE::Pspac-RBS[IPTG]-ywcD [spcR]	JVG12
76	JVG465	*Bacillus subtilis* 168	sacA::Pveg-eGFP [cmR], amyE::Pspac_low-RBS[IPTG]-ywcD [spcR]	JVG12
77	JVG328	*Bacillus subtilis* 168	ΔgalK::lox-kan-lox, sacA::Pveg-eGFP [spcR]	BKK38200
78	JVG329	*Bacillus subtilis* 168	ΔgalT::lox-kan-lox, sacA::Pveg-eGFP [spcR]	BKK38190
79	BSR1011	*Bacillus subtilis* NCIB 3610	pBS32 (ΔcomI), sacA::Pveg-eGFP [spcR]	DS7187**
80	BSR1021	*Bacillus subtilis* NCIB 3610	pBS32 (ΔcomI), sacA::Pveg-mRFP1 [spcR]	DS7187
81	BSR1031	*Bacillus subtilis* NCIB 3610	pBS32 (ΔcomI), sacA::Pveg-eGFP [spcR], swrA::lox-kan-lox	DS7187
82	VSG131	*Bacillus subtilis* NCIB 3610	pBS32 (ΔcomI), sacA::Pveg-eGFP [cmR], divIVA::lox-kan-lox	DS7187

*Parent strains with BKK and BKE come from genome-wide knockout library (1)
** ComI deletion is provided by Daniel Kearns (2)
*** Strain derived from (3)

Table S10. Integration vectors*

Identifier	Description	Snapgene File	Source
PL11	pECE174::Pveg-eGFP [spcR]	PL11_ECE174-Pveg-eGFP.dna	This study
PL12	pECE174::Pveg-mRFP1 [spcR]	PL12_ECE174-Pveg-mRFP1.dna	This study
PL13	pECE174::Pveg-eGFP [cmR]	PL13_ECE174-Pveg-eGFP.dna	This study
PL14	pECE174::Pveg-mRFP1 [cmR]	PL14_ECE174-Pveg-mRFP1.dna	This study
pDR244a	Plasmid with cre recombinase	pDR244.dna	(1)
pDRK110a	Inducible expression	pDRK110a.dna	(1)

*Snapgene files with details on plasmid history are provided in Supplementary Information

Table S11. Tn-seq samples

n	Sample	Description
1	S	Starting population
2	C1-a	Control 1: Starting population inoculated on recovery plate directly, replicate 1
3	C1-b	Control 1: Starting population inoculated on recovery plate directly, replicate 2
4	C2-a	Control 2: Starting population inoculated on recovery plate after adding *D. discoideum*
5	C3-a	Control 3: Starvation only (no LB, batch culture), replicate 1
6	C3-b	Control 3: Starvation only (no LB, batch culture), replicate 2
7	**NP**-N-a	Batch condition with no predation, replicate 1
8	**NP**-N-b	Batch condition with no predation, replicate 2
9	**NP**-N-c	Batch condition with no predation, replicate 3
10	**NP**-L-a	Batch conditions with low predation, replicate 1
11	**NP**-L-b	Batch conditions with low predation, replicate 2
12	**NP**-L-c	Batch conditions with low predation, replicate 3
13	**NP**-H-a	Batch condition with high predation, replicate 1
14	**NP**-H-b	Batch condition with high predation, replicate 2
15	**NP**-H-c	Batch condition with high predation, replicate 3
16	**NR**-N-a	Plate condition with no predation, replicate 1
17	**NR**-N-b	Plate condition with no predation, replicate 2
18	**NR**-N-c	Plate condition with no predation, replicate 3
19	**NR**-L-a	Plate condition with low predation, replicate 1
20	**NR**-L-b	Plate condition with low predation, replicate 2
21	**NR**-L-c	Plate condition with low predation, replicate 3
22	**NR**-H-a	Plate condition with high predation, replicate 1
23	**NR**-H-b	Plate condition with high predation, replicate 2
24	**NR**-H-c	Plate condition with high predation, replicate 3

Table S12. Tn-sequence data

n	Name	i7 barcode	i5 barcode	Tn-seq sample	Fastq files*
1	Ind1	ATCACG	CGATGT	S	Ind1_S15_L00**X**_R1_001.fastq.gz
2	Ind2	CGATGT	TTAGGC	C1-a	Ind2_S16_L00**X**_R1_001.fastq.gz
3	Ind3	TTAGGC	TGACCA	C1-b	Ind3_S17_L00**X**_R1_001.fastq.gz

4	Ind4	TGACCA	ACAGTG	C2-a	Ind4_S18_L00**X**_R1_001.fastq.gz
5	Ind5	ACAGTG	GCCAAT	C3-a	Ind5_S19_L00**X**_R1_001.fastq.gz
6	Ind6	GCCAAT	CAGATC	C3-b	Ind6_S20_L00**X**_R1_001.fastq.gz
7	Ind7	CAGATC	ACTTGA	**NP**-N-a	Ind7_S21_L00**X**_R1_001.fastq.gz
8	Ind8	ACTTGA	GATCAG	**NP**-N-b	Ind8_S22_L00**X**_R1_001.fastq.gz
9	Ind9	GATCAG	TAGCTT	**NP**-N-c	Ind9_S23_L00**X**_R1_001.fastq.gz
10	Ind10	TAGCTT	GGCTAC	**NP**-L-a	Ind10_S24_L00**X**_R1_001.fastq.gz
11	Ind11	GGCTAC	CTTGTA	**NP**-L-b	Ind11_S25_L00**X**_R1_001.fastq.gz
12	Ind12	CTTGTA	AGTCAA	**NP**-L-c	Ind12_S26_L00**X**_R1_001.fastq.gz
13	Ind13	AGTCAA	AGTTCC	**NP**-H-a	Ind13_S27_L00**X**_R1_001.fastq.gz
14	Ind14	AGTTCC	ATGTCA	**NP**-H-b	Ind14_S28_L00**X**_R1_001.fastq.gz
15	Ind15	ATGTCA	CCGTCC	**NP**-H-c	Ind15_S29_L00**X**_R1_001.fastq.gz
16	Ind16	CCGTCC	GTCCGC	**NR**-N-a	Ind16_S30_L00**X**_R1_001.fastq.gz
17	Ind18	GTCCGC	GTGAAA	**NR**-N-b	Ind18_S31_L00**X**_R1_001.fastq.gz
18	Ind19	GTGAAA	GTGGCC	**NR**-N-c	Ind19_S32_L00**X**_R1_001.fastq.gz
19	Ind20	GTGGCC	GTTTCG	**NR**-L-a	Ind20_S33_L00**X**_R1_001.fastq.gz
20	Ind21	GTTTCG	CGTACG	**NR**-L-b	Ind21_S34_L00**X**_R1_001.fastq.gz
21	Ind22	CGTACG	GAGTGG	**NR**-L-c	Ind22_S35_L00**X**_R1_001.fastq.gz
22	Ind23	GAGTGG	ACTGAT	**NR**-H-a	Ind23_S36_L00**X**_R1_001.fastq.gz
23	Ind25	ACTGAT	ATTCCT	**NR**-H-b	Ind25_S37_L00**X**_R1_001.fastq.gz
24	Ind27	ATTCCT	ATCACG	**NR**-H-c	Ind27_S38_L00**X**_R1_001.fastq.gz

*All samples were sequenced over 5 lanes (L004-L008) to get sufficient coverage, so each sample is associated with 5 fastq files that are named accordingly.

Table S13. Sequence data

n	Identifier	Forward read	Backward read
1	JVG98	JVG98_S199_R1_001.fastq.gz	JVG98_S199_R2_001.fastq.gz
2	JVG99	JVG99_S200_R1_001.fastq.gz	JVG99_S200_R2_001.fastq.gz
3	JVG100	JVG100_S201_R1_001.fastq.gz	JVG100_S201_R2_001.fastq.gz
4	JVG101	JVG101_S202_R1_001.fastq.gz	JVG101_S202_R2_001.fastq.gz
5	JVG102	JVG102_S203_R1_001.fastq.gz	JVG102_S203_R2_001.fastq.gz
6	JVG103	JVG103_S204_R1_001.fastq.gz	JVG103_S204_R2_001.fastq.gz
7	JVG104	JVG104_S205_R1_001.fastq.gz	JVG104_S205_R2_001.fastq.gz
8	JVG105	JVG105_S206_R1_001.fastq.gz	JVG105_S206_R2_001.fastq.gz
9	JVG106	JVG106_S207_R1_001.fastq.gz	JVG106_S207_R2_001.fastq.gz
10	JVG107	JVG107_S208_R1_001.fastq.gz	JVG107_S208_R2_001.fastq.gz
11	JVG141	JVG141_S209_R1_001.fastq.gz	JVG141_S209_R2_001.fastq.gz
12	JVG142	JVG142_S210_R1_001.fastq.gz	JVG142_S210_R2_001.fastq.gz
13	JVG143	JVG143_S211_R1_001.fastq.gz	JVG143_S211_R2_001.fastq.gz
14	JVG144	JVG144_S212_R1_001.fastq.gz	JVG144_S212_R2_001.fastq.gz
15	JVG145	JVG145_S213_R1_001.fastq.gz	JVG145_S213_R2_001.fastq.gz
16	JVG146	JVG146_S214_R1_001.fastq.gz	JVG146_S214_R2_001.fastq.gz
17	JVG147	JVG147_S215_R1_001.fastq.gz	JVG147_S215_R2_001.fastq.gz
18	JVG148	JVG148_S216_R1_001.fastq.gz	JVG148_S216_R2_001.fastq.gz
19	JVG149	JVG149_S217_R1_001.fastq.gz	JVG149_S217_R2_001.fastq.gz
20	JVG150	JVG150_S218_R1_001.fastq.gz	JVG150_S218_R2_001.fastq.gz
21	JVG151	JVG151_S219_R1_001.fastq.gz	JVG151_S219_R2_001.fastq.gz
22	JVG152	JVG152_S220_R1_001.fastq.gz	JVG152_S220_R2_001.fastq.gz
23	JVG153	JVG153_S221_R1_001.fastq.gz	JVG153_S221_R2_001.fastq.gz
24	JVG154	JVG154_S222_R1_001.fastq.gz	JVG154_S222_R2_001.fastq.gz
25	JVG155	JVG155_S223_R1_001.fastq.gz	JVG155_S223_R2_001.fastq.gz
26	JVG156	JVG156_S224_R1_001.fastq.gz	JVG156_S224_R2_001.fastq.gz
27	JVG157	JVG157_S225_R1_001.fastq.gz	JVG157_S225_R2_001.fastq.gz
28	JVG158	JVG158_S226_R1_001.fastq.gz	JVG158_S226_R2_001.fastq.gz
29	JVG159	JVG159_S227_R1_001.fastq.gz	JVG159_S227_R2_001.fastq.gz
30	JVG160	JVG160_S228_R1_001.fastq.gz	JVG160_S228_R2_001.fastq.gz
31	JVG161	JVG161_S229_R1_001.fastq.gz	JVG161_S229_R2_001.fastq.gz
32	JVG162	JVG162_S230_R1_001.fastq.gz	JVG162_S230_R2_001.fastq.gz
33	JVG163	JVG163_S231_R1_001.fastq.gz	JVG163_S231_R2_001.fastq.gz
34	JVG164	JVG164_S232_R1_001.fastq.gz	JVG164_S232_R2_001.fastq.gz
35	JVG165	JVG165_S233_R1_001.fastq.gz	JVG165_S233_R2_001.fastq.gz

Mutant variant mapping

Table S14. *Bacillus subtilis* 168

n	Identifier	Repl.	Independent*	Position	Gene	Locus tag	Type
1	JVG141	1	1	2,552,801	sinR→	BSU_24610	T→C, V50A (GTC→GCC)
2	JVG142	1	2	2,552,773	sinR→	BSU_24610	A→C, N41H (AAC→CAC)
3	JVG143	1	3	1,612,807	divIVA→	BSU_15420	C→T, A96V (GCG→GTG)
		1	4	2,029,033	yozQ→	BSU_18600	(A)7→8, coding (14/294nt)
4	JVG144	1	5	1,612,781	divIVA→	BSU_15420	(A)5→6, coding (261/495nt)
5	JVG145	1	-	2,552,801	sinR→	BSU_24610	T→C, V50A (GTC→GCC)
6	JVG146	1	6	1,612,798	divIVA→	BSU_15420	Δ28bp, coding (278-305/495nt)
7	JVG147	1	7	2,552,681	sinR→	BSU_24610	G→A, R10H (CGT→CAT)
8	JVG148	1	-	1,612,816	divIVA→	BSU_15420	(A)5→6, coding (296/495nt)
9	JVG149	1	-	2,552,801	sinR→	BSU_24610	T→C, V50A (GTC→GCC)
10	JVG150	1	-	NA	NA	NA	No detectable mutations

*Independent mutations

Table S15. *Bacillus subtilis* NCIB 3610

n	Identifier	Repl.	Independent*	Position	Gene	Locus tag	Type
1	JVG98	1	1	3,923,151	ywcC←/→slrA	B4U62_20565	G→A, intergenic (-148/-208)
2	JVG99	1	2	68,317		B4U62_22485	Δ243nt
3	JVG100	1	-	NA	NA	NA	No detectable mutations
4	JVG101	1	3	1,539,815	yktD←/←nprE	B4U62_08095	(T)7→8, intergenic (-100/+252)
			-	3,923,151	ywcC←/→slrA	B4U62_20565	G→A, intergenic (-148/-208)
5	JVG102	1	-	NA	NA	NA	No detectable mutations
6	JVG103	1	4	2,552,855	sinR→	B4U62_13305	T→C, L61S (TTG→TCG)
7	JVG104	1	5	2,552,969	sinR→	B4U62_13305	T→C, L99S (TTA→TCA)
8	JVG105	1	6	3,922,796	ywcC←	B4U62_20565	(T)8→7, coding (208/672nt)
9	JVG106	1	-	NA	NA	NA	No detectable mutations
10	JVG107	1	7	2,552,696	sinR→	B4U62_13305	A→C, Q8P (CAA→CCA)
11	JVG151	2	8	1,612,829	divIVA→	B4U62_08465	Δ28bp, coding (278-305/495nt)
			9	1,910,307	yndF→	B4U62_09670	A→G, T37A (ACA→GCA)
12	JVG152	2	10	2,552,906	sinR→	B4U62_13305	G→A, W78* (TGG→TAG)
13	JVG153	2	11	1,459,345	ptsG→/→ptsH	B4U62_07680	A→T, intergenic (+27/-71)
14	JVG154	2	12	1,612,993	divIVA→	B4U62_08465	T→C, W148R (TGG→CGG)
			13	3,950,545	ywaC←	B4U62_20700	Δ1bp, coding (79/633nt)
15	JVG155	2	-	NA	NA	NA	No detectable mutations
16	JVG156	2	14	1,612,849	divIVA→	B4U62_08465	New junction
17	JVG157	2	15	1,917,142	B4U62_09710–lexA	B4U62_09715	Δ1,170bp
18	JVG158	2	16	1,612,588	divIVA→	B4U62_08465	T→C, F13L (TTT→CTT)
19	JVG159	2	17	3,922,796	ywcC←	B4U62_20565	(T)8→7, coding (208/672nt)
			18	3,668,303		B4U62_19215	Δ367nt
20	JVG160	2	19	1,546,907	bipA→	B4U62_08135	T→C, R252R (CGT→CGC)
			20	1,918,140	lexA←	B4U62_09715	A→G, L50S (TTG→TCG)
			21	2,320,178	xpt←/←ypwA	B4U62_11995	T→A, intergenic (-122/+209)
21	JVG161	2	22	1,598,607	ftsZ→	B4U62_08400	C→T, L249F (CTT→TTT)
			23	1,598,695	ftsZ→	B4U62_08400	C→T, A278V (GCA→GTA)
22	JVG162	2	24	542,718	yddG→	B4U62_02850	A→G, G367G (GGA→GGG)
			25	1,612,642	divIVA→	B4U62_08465	C→T, Q31* (CAA→TAA)
23	JVG163	2	26	2,552,645	sinI→/→sinR	B4U62_13305	G→A, intergenic (+5/-29)
24	JVG164	2	27	69,509	divIC→	B4U62_00370	Δ4bp, coding (305-308/378nt)
			28	1,582,543	pbpB→	B4U62_08335	C→T, T189I (ACA→ATA)
			29	1,830,092	pksM→	B4U62_09355	(A)6→7, coding (8508/12789nt)
			30	2,132,446	ctpA←	B4U62_10675	C→T, S297N (AGC→AAC)
25	JVG165	2	31	3,621,524	[minJ]–[ctpB]	B4U62_18975	Δ1,407bp

*Independent mutations

Table S16. Mutational hotspots

Mutational hotspot	Gene name	Number of independent mutations in both *B. subtilis* 168 and NCIB 3610 strains
BSU_24610 / B4U62_13305	sinR	10 isolates, of which 8 unique mutations
BSU_15420 / B4U62_08465	divIVA	9 isolates, of which 8 unique mutations
BSU_38220 / B4U62_20565	slrC	4 isolates, of which 3 unique mutations
	Total	23 from the 35 isolates have mutation ()

42 mutations were observed among the 35 sequenced colonies, of which 10 colonies were sequenced from *B. subtilis* 168 and 25 were sequenced from *B. subtilis* NCIB 3610

Supplementary Text S1. Exploring the impact of population bottlenecks due to predation on count data.

Since predation results in strong population bottlenecks, where only few cells survive predation, it is expected to result in jackpot effects that cause strong variability between replicates. To explore this further, we performed numeric simulations. Using experimentally-determined mutant counts and cell counts in the starting population, we imposed bottlenecks where 0%, 90%, 99%, 99.9% or 99.99% of cells were cleared from the starting population by predation and subsequently simulated count data (see Methods). For simplicity, we ignored the role that growth could have on mutant abundances. Since we only found weakly enriched mutants in the nutrient-poor condition, we did not account for resistant mutant in our simulations. For the nutrient-rich conditions, we assumed about ~1% of the mutants was resistant (i.e., resistant mutants make up about 0.8% of all cells in the starting population; e.g., mutants above the diagonal in Fig. 2C).

By comparing depletion of mutants between our simulated count data and observed count data (Fig. S8), we can estimate the predation-imposed population bottleneck. For the nutrient-poor growth condition (NP), we estimate that 99 to 99.9% of the population was cleared due to predation (i.e., population bottleneck between 0.1% and 1%). This population bottleneck had a clear impact on the distribution of count data (Fig. S8B-D). Rather than observing a homogeneous distribution of counts, mutant counts followed a multi-modal distribution with four peaks. These peaks are linked to the number of replicates, where mutants are lost in all (first left peak), two (second peak), one (third peak) or none (i.e., fourth right peak) of the replicate populations. This multi-modal distribution highlights the severity of the population bottleneck. Performing similar simulations for the nutrient-rich growth conditions (NR), suggests that about 99.99% of the susceptible cells were consumed during predation (i.e., population bottleneck of 0.01%; Fig. 9). Also in this case, we find a multi-modal distribution of count data (Fig. 9B-D), although this is less apparent in our experimental count data, as most mutants have been fully depleted from the population.

For the nutrient-rich growth condition, we assumed that ~1% of mutants were resistant to predation. In the simulated count data, these resistant mutants show little variability in their counts across replicates and formed a clearly distinct and enriched cluster (Fig. S9A-B). In our experimental count data were resistant mutants (above diagonal in Fig. 2C and Fig. S9C) show considerable variability between replicates, despite being enriched. This suggests that mutants in our experiments are still partially susceptible to the population bottleneck, most likely because they are not fully resistant. Mutants might for example be

transiently resistance (i.e., for part of the growth curve) or just lower the predation probability. When mutants do not fully resistant to predation, their fate might still strongly differ between replicates, which should be accounted for when identifying resistant mutants. For that reason, we performed validation experiments for the most-enriched mutants, irrespective of their variability across replicates (see Data S1). This strategy proofed fruitful, because most validated mutants showed reproducible benefits in the context of predation (Fig. 3).

Supplementary Text S2. Competition assays with clean deletion mutants

We also performed the competition assays for clean deletion mutants, without antibiotic cassette, as – like the transposon inserts – also the antibiotic cassette in our knockout mutants can cause polar gene expression changes. Most of these competition assays gave similar results, with some quantitative differences (see Fig. 3 and Fig. S11): for example, for the *mnmE-trmF-rmsG* operon, clean deletions showed reduced benefits, while for Δ*minJ*, the clean deletion showed enhanced benefits under predation. An exception was Δ*tapA,* where we found the opposite results with and without the antibiotic cassette. With the antibiotic cassette, Δ*tapA* shows a strong competitive advantage under predation and without the cassette Δ*tapA* shows a competitive disadvantage. This suggests that the selective benefit of the original Δ*tapA* knockout results from the downstream overexpression of the Δ*tapA* operon. This finding is consistent with the observed fitness benefit of the Δ*sinR* knockout. SinR is the transcriptional repressor of *tapA*, so also Δ*sinR* overexpresses the *tapA* operon (4).

Supplementary Text S3. Detailed methods

All supplementary data files (Data S1-S4, Movies S1-S12) are available through Zenodo (https://doi.org/10.5281/zenodo.14871691).

Strains and culturing

For cloning and standard culturing, *Bacillus subtilis* 168 and *Bacillus subtilis* NCIB 3610, were grown on LB at 37°C (see Table S1-S10 for strain lists, consumables and media compositions). For selective plating of *B. subtilis*, antibiotics were used at the following concentrations: 7.5µg/ml kanamycin, 1µg/ml erythromycin + 12.5µg/ml lincomycin, 6µg/mL chloramphenicol, 100µg/mL spectinomycin. For *Escherichia coli*, ampicillin was used at 100µg/mL. For most predation experiments, *Dictyostelium discoideum* NC4 (DBS0304666; dictyBase) was precultured on LPB plates with *Escherichia coli* B/r (dictyBase DBS0305924). For time lapse movies and the predation assay with *B. subtilis* NCIB 3610, *Dictyostelium discoideum* AX2 (DBS0235521, dictyBase) or AX2 *act5*::*mCherry* (Table S9, Garriga-Canut et al. 2025) were used instead, which could be preculturing axenically. For

NR condition in the transposon screen, *D. discoideum* NC4 and *B. subtilis* 168 were grown on SM/5 plates. Plates were always freshly prepared: LPB plates were made by pouring 35mL of autoclaved LPB agar medium (Table S2) per Petri dish, drying plates 30 min at the flame and then inoculate cells according to experimental setup (see below). Similarly, SM and SM/5 plates were prepared by pouring 35mL of autoclaved SM and SM/5 agar medium (Table S3 and S4) per Petri dish, drying plates 30 min at the flame and then inoculating cells according to experimental setup (see below).

Transposon screen

The mariner Transposon library in *B. subtilis* 168 was kindly provided by Alan Grossman, and was generated as described in (5). The library was stored as a dense 200μL cell suspension with an estimated $\sim 2 \cdot 10^5$ mutants at -80°C. Before starting the predation assay, we thawed the library and resuspended cells in 20mL LB for 3 hours at 37°C (200mL Erlenmeyer flask, 250rpm), until they reached a density of ~$5 \cdot 10^8$ cells/mL (OD_{600}=1). Cells were estimated to have undergone ~3.3 divisions during preculturing, minimizing the impact of preculturing on mutant frequencies. After preculturing, cells were washed in 1x SorMC (Table S6) with a 2.5% (v/v) LB spike-in (Table S7), by centrifuging the cell suspension (6400xg, 4 min), removing supernatant and resuspending cells in SorMC buffer. This normalized cell suspension was subsequently used to start our predation assays, as further detailed below.

To prepare *D. discoideum*, cells were pre-cultured on LPB plates (Table S2) with *E. coli* B/r. In short, 100μL of dense *E. coli* B/r (Table S9) culture was spread on freshly-prepared LPB plates. After inoculation, plates were wrapped in parafilm and incubated upside down at 22°C overnight. The next day, *D. discoideum* NC4 cells were transferred from a feeding front of a previously-inoculated LPB plate to the center of the new LPB plates with *E. coli* B/r, using a 10μL inoculation loop. Plates were subsequently wrapped in parafilm and incubated at 22°C for 2 days. After 2 days, cells from feeding fronts on 8 LPB plates were scraped off using a 10μL inoculation loop and resuspended in 5mL of SorMC with 2.5% LB spike-in (15mL Falcon tube). *D. discoideum* cells were washed three times, by centrifuging cells (500xg, 4m), removing supernatant (4.5mL) and adding fresh SorMC buffer (4.5mL). Washing removed most of the *E. coli* B/r contamination. After washing, *D. discoideum* cells were counted using a disposable hemocytometer (Table S1) and normalized to either $6.25 \cdot 10^5$ cells/mL (low predation condition) or 10^7 cells/mL (high predation condition).

For both the nutrient-poor suspension (NP) and nutrient-rich agar plate condition (NR), we examined three predation levels, where the normalized *B. subtilis* cell suspension was mixed 50:50 with SorMC buffer only (i.e., without *D. discoideum*; N, no predation), with $6.25 \cdot 10^5$ *D. discoideum* cells/mL (L, low predation), or with 10^7 *D. discoideum* cells/mL (H, high predation). The SorMC buffer included a 2.5% LB spike-in to avoid nutrient stress, while minimizing *B. subtilis* growth. For the NP condition, we inoculated three wells per predation level (using a 24-well plate; Falcon, 353226) with 800µL cell suspension: 400µL of normalized *B. subtilis* cell suspension and 400µL of normalized *D. discoideum* cell suspension. The 24-well was incubated at room temperature (22-24°C), at the lowest shaking speed, while monitored optical density using a BioTek Synergy Plate Reader H4 (Reader Serial Number: 14042918). We incubated the 24-well plate until bacterial populations were cleared at the highest predation level (Fig. S3). All experiments were done in triplicates. As a control, we also included wells with normalized *B. subtilis* suspension in SorMC buffer without LB spike-in and without predator (C3), to solely monitor the impact of nutrient stress. This control had a similar mutant distribution as the low predation condition (see Fig. 2A), suggesting some changes in mutant counts can be attributed to nutrient stress. For the NR condition, we inoculated three SM/5 plates per predation level by spreading 200µL of normalized cell suspension across the plate (Table S4): 100µL of normalized *B. subtilis* cell suspension and 100µL of normalized *D. discoideum* cell suspension. Plates were dried at the flame for 10 min, wrapped in parafilm and incubated for 3 days at 22°C, until all replicate plates at the highest predation levels showed bacterial clearance (Fig. S4). All experiments were conducted in triplicates.

Since *B. subtilis* populations undergo a strong population bottleneck during predation, especially at high predation levels, we included a recovery step after predation to ensure we have sufficient biomass for library prep. This recovery step was applied to all conditions in the same way, to minimize any biases, including the no-predation conditions and control conditions. For the nutrient-poor condition, 100µL from each 800µL cell suspension was transferred to a recovery plate (i.e., LB agar plates), which was incubated for 5-6 hours at 37°C. Note that at these temperatures *D. discoideum* dies, so there is no predation during the recovery step. For the plate conditions, we scraped cells from the plate using an 10µL inoculation loop, resuspended cells in PBS suspension and plated (100µL) on recovery plates, which likewise were incubated for 5-6 hours at 37°C. After growth, cells were scraped from the recovery plates, resuspended in PBS and centrifuged (6500xg, 4 min). Supernatant was subsequently removed and pellets were flash frozen in liquid nitrogen and stored at -80°C.

We added two additional controls (C1 and C2) to assure that the recovery step did not bias our results. In one control (C1), we inoculated 100µL of normalized *B. subtilis* suspension from our starting culture on a recovery plate, which was incubated for 5-6 hours at 37°C. This control allows us to assess the impact of the recovery step on mutant counts and was done in duplicate. In another control (C2), we first mixed 100µL of normalized *B. subtilis* with 100µL 10^7 *D. discoideum* cells/mL and inoculated 200uL on a recovery plate. Similar to the other recovery plates, this plate was incubated for 5-6 hours at 37°C. This control was included to determine if *D. discoideum* could bias mutant counts during recovery step. Both controls confirmed that there was a minimal impact of the recovery plates on changes in mutant counts (Fig. S5).

Library preparation

Genomes were extracted from flash-frozen cell pellets (~1-2*10^9 cells) using Qiagen's DNeasy Blood and Tissue Extraction Kit (Qiagen, 69506), following manufacturer's protocol for gram-positive bacteria. The gDNA was subsequently digested using MmeI (NEB, R0637L). Following (5), for each sample, we mixed 4µg gDNA, 3µL MmeI (6 units; 2000 U/mL), 15µL of NEB rCutSmart buffer and dH_2O to 150µL total volume. We digested gDNA for 2.5h at 37°C, after which we added 1.2uL (6 units) quick CIP (5000 U/mL) and incubated samples for 20min at 37°C for phosphatase treatment and 10min at 80°C to inactivate the phosphatase. Digest was subsequently purified using QIAquick PCR Purification Kit (Qiagen, 28104) and eluted in 30µL 2mM Tris-Cl (pH8.5) buffer.

We subsequently annealed adapters (oCJ25 and oCJ26; Fig. S1 and Table S8), dissolved in dH_2O at 100µM, by mixing 35uL of each adapter and exposing this mixture 5min to 95°C and gradually letting it cool down to 20°C (50 cycles, -1.5°C/cycle, 1min/cycle). We then added 2µL of 100µM annealed adapter to 27.5µL of digested and dephosphorylated gDNA, 3.5µL 10x T4 DNA ligase buffer and 1.5µL (600 units) of T4 DNA ligase (400.000U/mL). Ligation happened overnight at 16°C. After ligation, ligation product was purified using QIAquick PCR Purification Kit and eluted in 50µL 2mM Tris-Cl (pH8.5) buffer.

5uL of ligation product was used for setting up a PCR. We mixed 5µL ligation product, 10µL 5xQ5 buffer, 1µL 10mM dNTP, 0.5uL Q5 polymerase, 2.5µL 10µM ocJ22-indX primer, 2.5µL 10µM ocJ23-indX primer and dH_2O up to a volume of 50uL. For each sample, another combination of primers was used to have unique barcodes combinations for Illumine sequencing (see Fig. S1 and Table S8, S11, and S12). The PCR reaction was repeated for 18 cycles (1m, 98°C; 18x [10s, 98°C; 30s, 68°C; 10s, 72°C], 2m, 72°C), after which the PCR product was purified and concentrated using the Zymo's DNA Clean & Concentrator-5 Kit

(Zymo, D4004), and eluted in 15µL EB buffer. After this, we performed a size selection using a Novex 8% TBE gel (Invitrogen, EC62152BOX) with the UTR ladder (Invitrogen, 10597012) as a reference, targeting the expected PCR product of 149bp (excising ±20bp). The excised gel was disrupted by centrifugation (16.000rpm, 3 min) through a needle-pierced 0.5µL LoBind Eppendorf tube and collected in a 1.5µL LoBind Eppendorf tube, after which the DNA was eluted in 400µL 1mM Tris (10m, 70°C, 1000rpm). DNA was subsequently purified and concentrated using Zymo's DNA Clean & Concentrator-5 Kit, and eluted in 15µL EB buffer. 1uL of eluted DNA was used to check the concentration and quality of the library prep using Agilent High Sensitivity DNA Kit (Agilent, 5067-4626) following manufacturer's instruction. Sequencing was performed using the Illumina HiSeq 4000 sequencing platform using single-end reads and custom sequencing primers (Fig. S1 and Table S8) at UCSF's Center for Advanced Technology (CAT). We had a total of 24 Tn-seq libraries that were sequenced over 5 lanes in a HiSeq 4000 flow cell, resulting in a total of 120 fastq files after demultiplexing (based on dual barcodes; see Table S12). All sequencing data is publicly available on the European Nucleotide Archive (ENA) database, accession number: PRJEB85855.

Analysis of Tn-seq screen

Reads in fastq files were mapped to the genome of *B. subtilis* 168 (RefSeq, GCF_000009045.1, NC_000964_3, Assembly ASM904v1) using Bowtie 1.3.1, converted into bam-files using Samtools and subsequently converted to wig-files using the Integrated Genome Viewer, IGVTools 2.4.19 (6). The distribution of raw reads is shown in Fig. S2. The per-nucleotide read counts are expected to be enriched close to the origin of replication in the genome, due to the genomic replication cycle. For each sample, we therefore corrected read counts for genome location by fitting a local polynomial regression across the genome (loess function in R4.2.2). Then we performed two types of mutant counts, either counting the number of reads for each 200bp window across the genome or counting the number of reads per genome feature (e.g., CDS). To avoid windows and features with low coverage in our starting library (i.e., few Tn-inserts), we removed windows and features with counts $<$ 5% of the median count values (for the window-based analysis, windows with less than 63 reads, for the feature-based analysis, features with less than 258 reads). Mutant counts were finally normalized across samples using the total mutant counts. The count matrices are provided in Data S1 for both the window- and feature-based analysis. We analyzed a total of 24 Tn-seq libraries (Data S1 and Table S11).

Mutant counts were compared across samples, using $\log_2(normalized\ count + 1)$, to determine mutants that were enriched under high predation. p values were determined using

a standard t test with unequal variance with a Benjamini-Hochberg correction for multiple testing. All statistical test results are provided in Data S1. In suspension (NP), there were many weakly but significantly enriched mutants (feature-based analysis, significantly-enriched features range from $0.9 < \log_2 FC < 3.7$; Data S1), while on plates (NR), mutants showed much stronger but often non-significant enrichment due to strong variability between replicates (feature-based analysis, significantly-enriched features range from $1.4 < \log_2 FC < 12.8$; Data S1). This variability is inherent to the plate assay, due to the severe population bottlenecks, like we observed for the low predation condition as well (NR-L in Fig. 2A). Given this drawback of the plate assay, we focused on the most-strongly enriched mutants for our validation experiments (Fig. 3), irrespective of their variability across replicates (see also Text S1). The fact that most validated mutants indeed showed a reproducible competitive benefit under predation confirms that enrichment alone is a useful measure for identifying genes relevant in predation evasion.

Simulations

The distribution of normalized counts from starting population were used to simulate the impact of predation and predation resistance on mutant counts before and after predation (see Fig. S8 and S9, Text S1). For the nutrient-poor condition, we used 400µL of $5 \cdot 10^8$ cells/mL in the experiment, so in our simulations we assume $2 \cdot 10^8$ cells are present (with $\sim 2 \cdot 10^5$ mutants, this implies a 1000x coverage of cells per transposon insertion site) at the start of predation, which are randomly sampled from the Tn-seq library. Then, after predation, we collected 100µL from the 800µL suspension for the recovery plates. Assuming that 0%, 90%, 99% or 99.9% or the cells are consumed, and none of the mutants are resistant to predation (i.e., we only find weak enrichment in Fig. 2B), we can simulate the expected mutant counts after predation by randomly sampling the surviving cells (Fig. S8A-B). Based on the simulated data, we estimate that between 99% and 99.9% of all susceptible cells were consumed in the nutrient-poor condition. Simulated data are in close agreement with the real data (Fig. S8C-D).

For nutrient-rich condition, we inoculate 100µL of bacterial cells on an agar plate (together with 100µL of *D. discoideum* suspension), which equals $5 \cdot 10^7$ bacterial cells in total (with $\sim 2 \cdot 10^5$ mutants, this implies a 250x coverage), so for our simulations we randomly sample the same number of cells from our starting Tn-seq library. Based on experiments, we estimate that ~99.99% of the cells are consumed during predation in the nutrient-rich condition and that approximately ~1% of the transposon mutants are resistant to predation (i.e., resistant mutants make up about 0.8% of all cells in the starting population; Fig. 2C).

Based on these parameters, we can simulate the expected mutant counts after predation by randomly sampling the surviving cells (Fig. S9A-B). Simulated data are in close agreement with the real data (Fig. S9C-D), although real data shows substantially more noise.

Spontaneous mutants leading the predation resistance

To screen for spontaneous mutants leading to predation resistance, we incubated both *B. subtilis* 168 and *B. subtilis* NCIB 3610 with *D. discoideum* for an extensive period of time, until resistant colonies emerged (~10 to 15 days). In short, 200µL of dense overnight culture ($> 10^9$ cells/mL) were mixed with 10^4 *D. discoideum* spores and plated on nutrient-rich SM plates. In total, one plate was prepared with *B. subtilis* 168 and two plates were prepared with *B. subtilis* NCIB 3610. Plates were wrapped in parafilm and incubated at 22°C until the first resistant colonies emerged that were sufficiently large for colony picking. This took approximately ~10 to 15 days. In total, 35 colonies were isolated from the different plates, which were subsequently propagated in LB at 37°C for both gDNA extraction and making glycerol stocks (Table S13, S14 and S15).

Genome extractions and mutant analysis

Following previous studies (7, 8), we extracted gDNA using Promega's Wizard Genomic DNA Purification Kit (Ref A1120, Promega) according to the manufacturer's instructions, with exception for the lysis step, were we resuspended cells in 20mg/mL of fresh lysozyme (L4919, Sigma) in 40mM EDTA solution for 60 min at 37°C (1000rpm). We determined the quantity and quality of gDNA samples using the NanoDrop 2000/2000c Spectrophotometer (ThermoFisher) and a 0.7% agarose (w/v) TBE gel. Library construction and whole-genome sequencing were performed by Microbial Genome Sequencing Center (MiGS, Pittsburg, USA; current renamed as SeqCenter). Genomes were sequenced using Illumina NextSeq 2000 platform with 151bp paired-end reads. All sequencing data are publicly available on the European Nucleotide Archive (ENA) database, accession number: PRJEB85855. Reads were mapped against associated reference genomes, *B. subtilis* 168 (RefSeq, GCF_000009045.1) and *B. subtilis* NCIB 3610 (RefSeq, GCF_002055965.1), using Breseq 0.35.4 (9, 10), and all identified mutants were subsequently evaluated manually using the Integrated Genome Browser, IGVTools 2.4.19 (6). Mutants are listed in Table S13, S14 and S15.

Strains and cloning

A full strain list of 82 strains used in this study is provided in Table S9. Information on oligos and integration plasmids are provided in Table S8 and S10 respectively. The transposon library was kindly provided by the lab of Alan Grossman (5). *D. discoideum* NC4, *D.*

discoideum AX2 and *E. coli* B/r were obtained from DictyBase (Table S9). The individual *B. subtilis* knockout mutants were obtained from the whole-genome knockout library of *B. subtilis* (11), strain numbers are specified in Table S9. For *B. subtilis* NCIB 3610, mutants were generated by transforming a Δ*comI* background strain (Table S9, Konkol et al. 2013) with the gDNA from a knockout strain of interest, obtained from the whole-genome knockout library (11), and plating on kanamycin selection plates. Mutants were confirmed through PCR.

Clean deletion. Clean deletions were generated using a Cre recombinase that removes antibiotic cassettes through its flanking *loxP* sites, following (11). In short, knockout strains were transformed with pDR244a, a temperature-sensitive plasmid with constitutive Cre recombinase expression, and plated on LB plates with spectinomycin at 30°C. Transformant colonies were subsequently re-streaked on LB plates and cured from their plasmid by overnight incubation at 45°C. Cells from the edge of those colonies were re-streaked again and loss of both the chromosomally-inserted antibiotic cassette and plasmid (pDR244a) were confirmed by both PCR and selective plating. The plasmid map of pDR244a is provided in Data S4.

Fluorescent strains. To create fluorescent strains, we transformed *B. subtilis* strains with one of several integration vectors (Table S10) targeting the *sacA* locus. The integration vectors were cloned using Gibson assembly (NEBuilder HiFi DNA Assembly Cloning kit). In short, ECE174 (*Bacillus Genetic Stock Center*, BGSC) was digested using EcoRV-HF and BamHI-HF, 37°C for 1h, followed DNA purification to remove the enzymes (QIAquick PCR purification kit). A spectinomycin and chloramphenicol antibiotic cassette were PCR-amplified using primers with appropriate overhangs for Gibson assembly (Table S8), using in-house plasmids as template (pJMP460 and ECE174 respectively). Gene fragments encoding fluorescent proteins (P_{veg}-*rfp* and P_{veg}-*gfp)* were ordered from IDT with appropriate overhangs for Gibson assembly. A two-fragment Gibson assembly was performed using the digested ECE174 plasmid and two gene fragments (a combination of the antibiotic cassette and fluorescent gene) resulting in four distinct integration vectors for the *sacA* locus: PL11 (P_{veg}-*gfp* [spcR]), PL12 (P_{veg}-*rfp* [spcR]), PL13 (P_{veg}-*gfp* [cmR]), and PL14 (P_{veg}-*rfp* [cmR]). Vectors were linearized using ScaI-HF and transformed into the appropriate mutant and wildtype *B. subtilis* strains following the high-throughput transformation protocol in (11). The plasmid maps of PL11, PL12, PL13 and PL14 are provided in Data S4.

Overexpression. Overexpression vectors were created through restriction-ligation, using pDRK110a – an integration vector with IPTG-inducible promoter and *lacI* repressor. First,

genes/operons of interest were PCR-amplified using primers specified in Table S8. pDRK110a and PCR products were subsequently digested using SphI-HF and SalI-HF, 1h at 37°C, followed by heat inactivation of the enzymes for 20min at 80°C. Plasmid digest was treated with quick CIP (NEB, M0525S) for 20min at 37°C and 20min at 80°C (inactivation of phosphatase). Digestion products were purified and then ligated overnight at 16°C, using T4 DNA Ligase (NEB, M0202) following manufacturer's instructions. *B. subtilis* was transformed using the integration vectors and double crossover recombination in the *amyE* locus was confirmed using starch plates and PCR. Depending on the gene/operon of interest, either the native Shine–Dalgarno sequence was used for cloning, or an optimized Shine–Dalgarno sequence from the IPTG-inducible promoter was used to maximize expression (12). Overexpression was achieved through IPTG induction with 1mM IPTG. The plasmid map of pDRK11a is provided in Data S4. Fig. S12 shows the competition assays with the overexpression strains: half the overexpression mutants show a competitive benefit under predation.

Competition assays for validating NR transposon hits

To perform scalable competition assays on SM/5 agar, we used an adjusted protocol based on 24-well plates (Fig. 3, 6, 7, S11, and S12). To each well, we added 2mL of SM/5 agar medium supplemented with 50µg/mL spectinomycin, to avoid growth of *E. coli* B/r that might come from the preculture condition of *D. discoideum* NC4. For overexpression mutants (Fig. S12), we also added 1mM IPTG (final concentration). The wells in the 24-well plate function as miniature agar plates, so like for the actual agar plates in our transposon screen, we dried the 24-well plate for 30min at the flame. Before inoculation, *D. discoideum* and *B. subtilis* are precultured in the same way as for the transposon screen. To have the same ratio between *B. subtilis* and *D. discoideum* cells as in the transposon screen (i.e., high predation condition), while maintaining approximately the same density of cells per surface area, we normalize the *B. subtilis* cell suspension to 10^8 cells/mL and *D. discoideum* to 10^7 cells/mL in SorMC medium. For *B. subtilis*, 10% of the cells were derived from the mutant strain (*gfp* expressing) and 90% from a wildtype strain (*rfp* expressing). Mutants were derived from the whole-genome knockout library of *B. subtilis* (11), after integrating P_{veg}-*gfp* into the *sacA* locus (see Methods above). We confirmed that the expression of the fluorescent reporters had no fitness effect (Fig. 3A). We mixed *D. discoideum* and *B. subtilis* cell suspension 50:50 and inoculated 30µL to each well, which we spread over the agar surface by tilting the plate. For the condition without predation, *B. subtilis* cell suspension was mixed 50:50 with SorMC buffer only. After inoculating the wells, the plate was dried for another 30min at the flame, wrapped in parafilm and incubated at 22°C. Like for the transposon library, cells were incubated for 3 days.

After competition, 1mL LB medium was added to each well in the 24-well plate, and cells were scraped from the surface into suspension using a 10µL inoculation loop. 10µL of this cell suspension was transferred to a 96-well plate with 190µL LB medium per well (supplemented with 50µg/mL spectinomycin). Cell were allowed to recover for 2.5h at 37°C, similar to the recover step in our transposon screen. Since cells suspensions were not normalized before the recovery step, some population recovered quicker than others, we therefore measured the optical density across the entire plate after growth using the BioTek Synergy Plate Reader H4. By including reference wells with LB only, or normalized cells suspensions with an OD_{600} of 0.1 ($5 \cdot 10^7$ cells/mL), 0.01 ($5 \cdot 10^6$ cells/mL) and 0.001 ($5 \cdot 10^5$ cells/mL) respectively, we could ensure that none of the populations were overgrown before analyzing them using the flow cytometer. 30µL of the recovered cell suspensions were subsequently transferred to a 96-well plate (with V-shaped bottoms) with 220µL PBS buffer per well, resulting in a final cell density between $5 \cdot 10^5$ and $5 \cdot 10^6$ cells/mL. This diluted plate was used for counting the numbers of *rfp*- and *gfp*-expressing cells in each well using high-throughput flow cytometry (BD LSR II Flow Cytometer).

We used the same high-throughput flow cytometry settings for all sampling. In short, before acquiring sample, the flow cytometer mixes a cell suspension four times, with a mixing volume of 100µL and a mixing speed of 200µL/sec, then it samples 50µL at a flow rate of 1µL/sec, which depending on the well (e.g., control and cell suspension) results in 100 to 10,000 events/sec. The following gains (voltages) were applied for the acquisition parameters: FSC (forward scatter), 500; SSC (side scatter), 350; FITC (*gfp* expression), 650; PE-Texas Red (*rfp* expression), 630. Events were only recorded with a minimal threshold of FSC = 500 and SSC = 500. After acquisition, we gated the events based on FSC and SSC to discriminate between debris and cells: $\log_{10}(FSC_{Height}) > 3$ and $\log_{10}(SSC_{Height}) > 3.75$. For the gated events, we counted the number of *gfp*-expressing cells ($\log_{10}(GFP_{Height}) > 3.5$) and *rfp*-expressing cells ($\log_{10}(RFP_{Height}) > 3.5$) from which we calculated the fraction of wildtype cells (see Data S1). See Fig. S10 for gating procedure. All flow cytometry data is publicly available through Zenodo (10.5281/zenodo.14871691).

Competition assays in suspension

For the competition assays with *B. subtilis* NCIB 3610 Δ*swrA*, predation resistance was quantified in suspension, where we could directly measure predation resistance using flow cytometry after predation (without recovery step). Like for the experiments above, wildtype and mutant strains with complementary fluorescent reporters were mixed 90:10 and exposed

to predation by *D. discoideum*. To avoid contamination of *E. coli* B/r from pre-culturing, we used the axenic *D. discoideum* AX2 strain, instead of NC4.

B. subtilis cells from an overnight culture were diluted to OD_{600} of 0.01 in either 5mL LB in culture tubes (for mutants) or 100mL LB in a 500mL flask (for wildtype cells). Culture tubes were incubated at 37°C (60rpm) on a roller drum and flasks at 37°C (100rpm) in a regular incubator until reaching an OD_{600} of 1 (~3h). For mutants, cells were harvested by centrifugation at 2000xg (1 min), supernatant was carefully removed without disturbing the cell pellet, and 400µL of KK2-MC containing 2.5% LB (referred as KK2-MC-LB) was added to each tube. Cells were carefully resuspended by softly flicking the tube, and cell concentration measured by OD_{600}. The concentration was adjusted to OD_{600} of 2 with KK2-MC-LB. For the wildtype, cells were transferred to 2x50mL tubes, centrifugated at 4000xg for 5 min, washed once in KK2-MC-LB, and then resuspended in 1mL KK2-MC-LB. OD_{600} was measured and cell concentration adjusted to OD_{600} of 2.

D. discoideum AX2 cells were grown on 4xSM/5-agar plates for 3 days at 22°C. The feeding fronts of all 4 plates were harvested with a loop and pooled together, washed 3 times with 1mL of HL5-FAB (HLG0102, ForMedium) (3), and cell concentration was measured with an automated cell counter (Countess III, Thermo Fisher). Cells were diluted to 10^5 cells/mL in 100mL HL5-FAB and grown at 22°C for 2 days (180rpm), until reaching exponential phase (0.8-1.2x10^6 cells/mL). Cells were harvested by centrifugation at 400xg (5 min), washed three times with KK2-MC-LB, resuspended in 1mL of KK2-MC-LB, and the concentration was adjusted to 10^7 cells/mL in KK2-MC-LB.

Using an Eppendorf's Multipette E3x multi-dispenser pipette, the following components were added to each well of a 24 well plate: 300µL of normalized *D. discoideum* AX2 cells, 270µL of normalized wildtype *B. subtilis* cells and 30µL of normalized mutant cells (either Δ*swrA* or Δ*divIVA*). For non-predation control wells, the volume of *D. discoideum* AX2 was replaced by 300µL KK2-MC-LB media. Multi-well plate were covered with an Breathe-Easy sealing membrane (Merch Z380059) and incubated at 22°C (300rpm) for 2-2.5h. To stop predation, *D. discoideum* AX2 cells were killed by incubating the plate at 37°C for 30 min (300rpm).

For non-predation control wells, a 10µL aliquot, and for predation wells, 50µL aliquot were transferred to the 96 well plate. Volume was adjusted to 200µL with KK2-MC buffer and analyzed by flow cytometry in an Attune CytPix Flow Cytometer with an CytKick Autosampler with the following settings: FSC (forward scatter), 350V; SSC (side scatter), 350V; BL1 (*gfp* expression), 383V; YL1 (*rfp* expression), 346V. Gating for bacteria was done manually using

FSC and SSC, and fluorescent intensities were used to determine the fraction of wildtype and mutant cells inside the population following Fig. S10.

Microscopy

To visualize single cell phenotypes of *B. subtilis* wildtype and resistant mutants, we performed widefield microscopy using agarose pads. *B. subtilis* was grown overnight in 3 ml LB grown at 37°C (220rpm) and back diluted in 3ml LB at an OD_{600} of 0.01. For most mutants, cells were harvested after 3 to 5h of regrowth at 37°C (220rpm) at an OD_{600} of 1 to 2. For Δ*slrC* and Δ*tapA*, cells were back diluted twice and cultured overnight under static growth conditions in a 24-well plate containing 1ml LB at 37°C without shaking. Compared to wildtype, Δ*slrC* and Δ*tapA* mutants showed strong filamentation and aggregation under static growth, but aggregates broke apart under shaken conditions.

Agarose pads were prepared by pipetting 300-500µl buffer on a glass slide (1% agarose, 10% LB, 90% KK2) and flattening it with another glass slide until it solidified. For shaken *B. subtilis* cell suspensions, 1mL bacterial cells were concentrated by centrifugation (8000xg, 2 min) and removing ~90% of supernatant. 5µL of bacterial suspension was pipetted onto the agarose pad. For static culture, the pellicle was scooped up with a blue inoculation loop and dipped on the agarose pad. The agarose pad was sealed with a coverslip and imaged upside-down. Imaging was performed on a Nikon Ti2-E inverse microscope using a 100x Oil objective (MRD31905) in widefield mode with phase contrast (Ph3). Exposure time was kept at 60ms and intensity at 20% or 70%. Most images were cropped to a size of 97.5µm x 97.5µm and contrast was adjusted visually. In all images, scale bars equal 10µm.

For the NR condition, populations of transposon mutants were cryopreserved as glycerol aliquots after the transposon screen for further examination later. For Fig. 4B, these aliquots from both the NR-N and NR-H conditions were recovered in LB cultures overnight (37°C) and precultured by back-diluting to an OD_{600} of 0.01 like described above. Early-stationary cells were concentrated (6.500xg, 4 min) and imaged using agarose pads. Approximately 20 images were taken per conditions and analyzed using Fiji. Each cell and filament in focus was manually measured by drawing a line along the major axis from the outermost poles. Images are shown in Fig. S13 and measurements are provided in Data S1.

For time lapse movies with predation, *D. discoideum* AX2 *act5*::*mCherry* was precultured on an SM/5 plate with a lawn of *E. coli* B/r at 22°C. After 3 to 5 days, cells were harvested from the feeding front and resuspended in 1mL KK2. Cells were washed three times by centrifugation at 400xg (4 min) and resuspending in KK2. Cells were subsequently counted

(CountessIII, Thermo Fisher). A Petri-dish with 20mL HL5 medium (HLG0102, ForMedium) was inoculated with 10^5 cells/mL and grown for two days at 22°C until exponential phase. Cells were harvested, washed once by centrifuging at 400xg (4 min), resuspended in KK2 buffer and normalized to a cell density of 10^6 cells/ml. 50µL of this cell suspension was pipetted onto a coverslip and allowed to settle for 15 min. The residual liquid was removed from the coverslip and the coverslip was placed upside down onto the agarose pad and imaged like described above. Time lapses were typically acquired by imaging every minute for a period of 1 to 2h.

Phase variation

To identify the prevalence of phase variation due to slipped strand mispairing of 8-mer repeats, we compared all genomes of *Bacillus subtilis* with the highest assembly levels available on March 2021 (388 whole-genome sequences of *B. subtilis* strains in total; Data S1) to our reference genome of *Bacillus subtilis* 168 (RefSeq, GCF_000009045.1, NC_000964_3, Assembly ASM904v1). We started by identifying orthologs using bidirectional best blast hits (BBH). To include nearby orthologs for our analysis only, we trimmed the list of BBH to only include orthologs with a nucleotide alignment similarity of >99% of the maximum alignment score (Global Smith-Waterman alignment; with 2 additional points for nucleotide match, -1 penalty for a nucleotide mismatch, -0.5 penalty a gap, and -0.1 penalty for extending gap). For each of these close orthologs, we screened for 8-mer repeats consisting of single, double, triple, quadruple or penta-nucleotide repeats. When a gene in the reference genome has an 8-mer repeat (e.g., *slrC*), while the ortholog shows the same repeat that is either shorter or longer, we determined if the altered repeat caused a frameshift by aligning the amino acid sequences downstream of the repeat between the reference and target genes (including 20 amino acids downstream of the repeat). If there is a frameshift, we concluded that indels in the nucleotide repeat are associated with phase variation. Fig. 8 shows the percentage of frameshifts observed in orthologs of genes with 8-mer repeats. For *slrC*, 2.6% of the orthologs have a frameshift mutation due to an indel in the 8-mer repeat. For comparison, a gene with known phase variation, *swrA*, has 15.5% of orthologs with a frameshift, while for *sinR* and *divIVA* (which do not have 8-mer repeats) frameshift mutations were not observed. This suggests that the small nucleotide repeat in *slrC* supports switch-like adaptations through phase variation.

References

1. B. M. Koo, *et al.*, Construction and analysis of two genome-scale deletion libraries for *Bacillus subtilis*. *Cell Syst* **4**, 291-305.e7 (2017).

2. M. A. Konkol, K. M. Blair, D. B. Kearns, Plasmid-encoded ComI inhibits competence in the ancestral 3610 strain of *Bacillus subtilis*. *J Bacteriol* **195**, 4085–4093 (2013).

3. M. Garriga-Canut, *et al.*, Selection-free CRISPR-Cas9 editing protocol for distant *Dictyostelid* species. [Preprint] (2025). Available at: https://www.biorxiv.org/content/10.1101/2025.03.23.644600v1 [Accessed 23 May 2025].

4. F. Chu, D. B. Kearns, S. S. Branda, R. Kolter, R. Losick, Targets of the master regulator of biofilm formation in *Bacillus subtilis*. *Molecular Microbiology* **59**, 1216–1228 (2006).

5. C. M. Johnson, A. D. Grossman, Identification of host genes that affect acquisition of an integrative and conjugative element in Bacillus subtilis. *Mol Microbiol* **93**, 1284–1301 (2014).

6. H. Thorvaldsdóttir, J. T. Robinson, J. P. Mesirov, Integrative Genomics Viewer (IGV): high-performance genomics data visualization and exploration. *Briefings in Bioinformatics* **14**, 178–192 (2013).

7. J. van Gestel, M. Ackermann, A. Wagner, Microbial life cycles link global modularity in regulation to mosaic evolution. *Nat Ecol Evol* **3**, 1184–1196 (2019).

8. J. van Gestel, A. Wagner, M. Ackermann, Pleiotropic hubs drive bacterial surface competition through parallel changes in colony composition and expansion. *PLOS Biology* **21**, e3002338 (2023).

9. J. E. Barrick, *et al.*, Identifying structural variation in haploid microbial genomes from short-read resequencing data using *breseq*. *BMC Genomics* **15**, 1039 (2014).

10. D. E. Deatherage, J. E. Barrick, “Identification of mutations in laboratory-evolved microbes from next-generation sequencing data using *breseq*” in *Engineering and Analyzing Multicellular Systems: Methods and Protocols*, Methods in Molecular Biology., L. Sun, W. Shou, Eds. (Springer New York, 2014), pp. 165–188.

11. B. M. Koo, *et al.*, Construction and analysis of two genome-scale deletion libraries for *Bacillus subtilis*. *Cell Syst* **4**, 291-305.e7 (2017).

12. S. Guiziou, *et al.*, A part toolbox to tune genetic expression in *Bacillus subtilis*. *Nucleic Acids Research* **44**, 7495–7508 (2016).
